# Supplementary material for: Adaptation, phylogeny, and covariance in milk macronutrient composition
Source: PeerJ. 2019 Nov 13;7:e8085. doi: 10.7717/peerj.8085 (PMC6858816; doi:10.7717/peerj.8085)
Supplement: Supplemental Information 1 [file peerj-07-8085-s001.zip › adaptation-phylogeny-covariance-milk-composition.html]

Introduction


# Introduction

This is an `R` script to accompany the PeerJ manuscript:
“Adaptation, phylogeny, and covariance in milk macronutrient
composition” (Article ID: 34219)

All the code can be run with results embedded in the output html file
with `rmarkdown::render()` or `knitr::spin()`.

# `R` Dependencies

A version of the `ksi` package allowing 2- and 3-variate tests is
available on gitlab and can be installed with `devtools`.
`devtools::install_gitlab("gregblomquist/ksi")`
Linux (e.g. Debian, Ubuntu) users may need to install `gsl-bin`
and `libgsl-dev` through their `apt` package manger for `ksi` to
install properly.

A few helper functions for plotting or making tables are included
in the scripts directory. All other *packages* are available on CRAN
or are installed by default with `R`.

```
library("ksi")
library("MCMCglmm")
```

```
## Loading required package: Matrix
```

```
## Loading required package: coda
```

```
## Loading required package: ape
```

```
library("PhylogeneticEM")
source("scripts/encl.R")
source("scripts/ggphylomorpho.R")
source("scripts/gtablephylo4d.R")
library("phytools")
```

```
## Loading required package: maps
```

```
library("ggplot2")
library("gridExtra")
library("reshape2")
library("phylobase")
```

```
## 
## Attaching package: 'phylobase'
```

```
## The following object is masked from 'package:ape':
## 
##     edges
```

```
library("adephylo")
```

```
## Loading required package: ade4
```

```
library("viridis")
```

```
## Loading required package: viridisLite
```

```
library("png")
library("grid")
```

# Raw Data

The raw data are provided in the `data` directory. This consists
of the final cleaned milk composition and ecological data and a
consensus phylogeny.

```
milk <- read.csv("data/milkData.csv")
rownames(milk) <- milk$phyName
milk$Diet <- factor(milk$Diet, levels = c("herbivore", "omnivore", "carnivore")) ## better order v alphabetical
summary(milk)
```

```
##                     phyName              Order                Family  
##  Acomys_cahirinus       :  1   Artiodactyla :23   Bovidae        :13  
##  Alces_alces            :  1   Carnivora    :23   Cervidae       : 7  
##  Alopex_lagopus         :  1   Primates     :20   Otariidae      : 7  
##  Alouatta_palliata      :  1   Rodentia     :15   Phocidae       : 7  
##  Alouatta_seniculus     :  1   Chiroptera   :10   Cercopithecidae: 6  
##  Arctocephalus_australis:  1   Diprotodontia: 9   Lemuridae      : 5  
##  (Other)                :118   (Other)      :24   (Other)        :79  
##                     Species          N      Lactation.stage
##  Acomys cahirinus       :  1   4      :13   150    :  3    
##  Alces alces            :  1   3      :11   30-60  :  3    
##  Alouatta palliata      :  1   6      :10   Mid?   :  3    
##  Alouatta seniculus     :  1   5      : 8   ~210   :  2    
##  Arctocephalus australis:  1   7      : 8   14-35  :  2    
##  Arctocephalus gazella  :  1   8      : 5   15-20  :  2    
##  (Other)                :118   (Other):69   (Other):109    
##    Dry.matter         Fat           Protein           Sugar      
##  Min.   : 8.80   Min.   : 0.20   Min.   : 1.100   Min.   : 0.02  
##  1st Qu.:16.52   1st Qu.: 4.75   1st Qu.: 4.175   1st Qu.: 3.00  
##  Median :22.75   Median : 8.75   Median : 6.950   Median : 4.80  
##  Mean   :27.27   Mean   :14.50   Mean   : 6.787   Mean   : 4.93  
##  3rd Qu.:32.27   3rd Qu.:19.23   3rd Qu.: 9.425   3rd Qu.: 6.60  
##  Max.   :71.10   Max.   :61.10   Max.   :15.800   Max.   :14.00  
##  NA's   :6                                        NA's   :15     
##        FP              FPS            itFat           itProtein     
##  Min.   : 1.600   Min.   : 8.20   Min.   :-6.2126   Min.   :-4.499  
##  1st Qu.: 9.675   1st Qu.:15.80   1st Qu.:-2.9985   1st Qu.:-3.133  
##  Median :15.750   Median :20.50   Median :-2.3446   Median :-2.594  
##  Mean   :21.284   Mean   :24.49   Mean   :-2.2538   Mean   :-2.794  
##  3rd Qu.:29.900   3rd Qu.:27.60   3rd Qu.:-1.4356   3rd Qu.:-2.263  
##  Max.   :69.000   Max.   :67.00   Max.   : 0.4515   Max.   :-1.673  
##                   NA's   :15                                        
##     itSugar        Female.mass        Gestation      Lactation.length
##  Min.   :-8.517   Min.   :-3.0963   Min.   : 0.420   Min.   : 0.300  
##  1st Qu.:-3.476   1st Qu.:-1.0479   1st Qu.: 1.810   1st Qu.: 1.675  
##  Median :-2.987   Median :-0.2165   Median : 5.480   Median : 4.500  
##  Mean   :-3.240   Mean   : 0.0000   Mean   : 5.846   Mean   : 6.164  
##  3rd Qu.:-2.650   3rd Qu.: 1.0886   3rd Qu.: 8.398   3rd Qu.: 8.350  
##  Max.   :-1.815   Max.   : 4.2311   Max.   :21.460   Max.   :42.000  
##  NA's   :15                                                          
##  RelLact.length      Litter.mass         Repro.output     
##  Min.   :-1.08369   Min.   :      0.3   Min.   :-3.17143  
##  1st Qu.:-0.07267   1st Qu.:     48.0   1st Qu.:-0.04649  
##  Median : 0.03426   Median :    457.5   Median : 0.25454  
##  Mean   : 0.00000   Mean   :  55092.1   Mean   : 0.00000  
##  3rd Qu.: 0.13967   3rd Qu.:   8707.8   3rd Qu.: 0.47344  
##  Max.   : 0.33039   Max.   :2272500.0   Max.   : 1.05042  
##                                                           
##  Developmental.stage.at.birth        Diet         Arid       
##  Min.   :0.000                herbivore:60   Min.   :0.0000  
##  1st Qu.:1.000                omnivore :32   1st Qu.:0.0000  
##  Median :2.000                carnivore:32   Median :0.0000  
##  Mean   :1.855                               Mean   :0.2823  
##  3rd Qu.:3.000                               3rd Qu.:1.0000  
##  Max.   :3.000                               Max.   :1.0000  
##                                                              
##     Aquatic           ordAq       
##  Min.   :0.0000   Min.   :0.0000  
##  1st Qu.:0.0000   1st Qu.:0.0000  
##  Median :0.0000   Median :0.0000  
##  Mean   :0.1774   Mean   :0.4113  
##  3rd Qu.:0.0000   3rd Qu.:0.0000  
##  Max.   :1.0000   Max.   :3.0000  
##
```

```
nrow(milk)
```

```
## [1] 124
```

```
consTree <- read.tree("data/consTree.phy")
consTree
```

```
## 
## Phylogenetic tree with 124 tips and 123 internal nodes.
## 
## Tip labels:
##  Arctocephalus_australis, Neophoca_cinerea, Arctocephalus_gazella, Arctocephalus_pusillus, Arctocephalus_tropicalis, Zalophus_californianus, ...
## 
## Rooted; includes branch lengths.
```

# Phylomorphospace Plots and Nutritional Geometry

Categories clades for coloring points in plots.

```
milk$group <- rep(NA, nrow(milk))
milk$group[milk$Order %in% c("Monotremata")] <- 1
milk$group[milk$Order %in% c("Dasyuromorphia","Didelphimorphia","Diprotodontia","Peramelemorphia")] <- 2
milk$group[milk$Order %in% c("Proboscidea")] <- 3
milk$group[milk$Order %in% c("Soricomorpha")] <- 4
milk$group[milk$Order %in% c("Artiodactyla","Cetacea")] <- 5
milk$group[milk$Order %in% c("Carnivora")] <- 7
milk$group[milk$Order %in% c("Chiroptera")] <- 6
milk$group[milk$Order %in% c("Perrissodactyla")] <- 8 # spelled wrong in data file, leave it here
milk$group[milk$Order %in% c("Primates")] <- 9
milk$group[milk$Order %in% c("Lagomorpha")] <- 10
milk$group[milk$Order %in% c("Rodentia")] <- 11
milk$group <- factor(milk$group, levels=1:11, labels=c("monotremes","marsupials","elephants","shrew","cetartiodactyls","bats","carnivores","perissodactyls","primates","rabbits","rodents"))
table(milk$group, useNA="ifany")
```

```
## 
##      monotremes      marsupials       elephants           shrew 
##               2              12               2               1 
## cetartiodactyls            bats      carnivores  perissodactyls 
##              29              10              23               7 
##        primates         rabbits         rodents 
##              20               3              15
```

```
gfpsp <- ggphylomorpho(tree=consTree, tipinfo=milk, xvar=Protein, yvar=Fat, title=NULL, xlab="Protein", ylab="Fat", labelvar=phyName, factorvar=group, shapevar=factor(milk$Aquatic), repel=FALSE, edge.width=.6, tree.alpha=.4, point.size=4, label.tips=FALSE) + theme_classic() + theme(legend.position=c(.15,.7)) + guides(color=guide_legend(title=""), shape=FALSE) ## stored for writing to disk in code below
```

```
## Loading required package: ggrepel
```

Create percentages of the fat+protein+sugar sums for nutritional geometry right-angle mixture model.
Plot fat vs. protein with sugar as the constrained diagonal isoclines.

```
milk$Fatr <- 100* milk$Fat / milk$FPS
milk$Proteinr <- 100* milk$Protein / milk$FPS
```

Have to drop the species missing sugar.

```
summary(milk[,c("Fatr","Proteinr")])
```

```
##       Fatr           Proteinr     
##  Min.   : 2.439   Min.   : 7.313  
##  1st Qu.:30.000   1st Qu.:19.708  
##  Median :42.512   Median :27.664  
##  Mean   :43.660   Mean   :27.241  
##  3rd Qu.:56.140   3rd Qu.:34.337  
##  Max.   :91.194   Max.   :49.789  
##  NA's   :15       NA's   :15
```

```
subTree <- drop.tip(consTree, as.character(milk$phyName[is.na(milk$Fatr)]) )
subDat <- subset(milk, is.na(milk$Fatr)==FALSE)

gfpsng <- ggphylomorpho(tree=subTree, tipinfo=subDat, xvar=Proteinr, yvar=Fatr, title=NULL, xlab="Protein", ylab="Fat", labelvar=phyName, factorvar=group, shapevar=factor(subDat$Aquatic), repel=FALSE, edge.width=.6, tree.alpha=.4, point.size=4, label.tips=FALSE) + theme_classic() + theme(legend.position=c(.85,.8)) + guides(color=FALSE, shape=FALSE) + xlim(c(5,55)) + ylim(c(0,100)) + geom_abline(slope=-1,intercept=50,linetype=2,alpha=.5) + geom_abline(slope=-1,intercept=25,linetype=2,alpha=.5) + geom_abline(slope=-1,intercept=75,linetype=2,alpha=.5) + geom_abline(slope=-1,intercept=100,linetype=2,alpha=.5)
```

```
gphylomorphnutr <- grid.arrange(gfpsp, gfpsng, nrow=1, ncol=2) ## prints to embed
```

```
ggsave(gphylomorphnutr, filename = "output/gPhylomorphoNutrGeom.pdf", height=6, width=12) ## for manuscript
```

# KSI Distinctive Clade Tests

Do some node labeling to ease interpretation.

```
consTreeLab <- consTree
consTreeLab$node.label <-paste0("nd", seq_len(consTree$Nnode))

labDat <- read.table(text="monotremes  Ornithorhynchus_anatinus Tachyglossus_aculeatus
marsupials   Macropus_rufus Didelphis_virginiana
diprotodonts Macropus_rufus Phascolarctos_cinereus
macropodiforms Macropus_rufus Bettongia_gaimardi
placentals Panthera_leo Loxodonta_africana 
carnivores Panthera_leo Arctocephalus_australis 
canids Vulpes_vulpes Nyctereutes_procyonoides
arctoids Neovison_vison Arctocephalus_australis
musteloids Neovison_vison Mephitis_mephitis
ursids  Ursus_arctos Ursus_americanus 
pinnipeds  Leptonychotes_weddellii Arctocephalus_australis
phocids Leptonychotes_weddellii Phoca_vitulina 
otariids Callorhinus_ursinus Arctocephalus_australis
Arctocephalus Arctocephalus_australis Arctocephalus_tropicalis
Mirounga Mirounga_leonina Mirounga_angustirostris
primates Gorilla_beringei Eulemur_mongoz
anthropoids Miopithecus_talapoin Alouatta_palliata
catarrhines Miopithecus_talapoin Gorilla_beringei
cercopithecoids Miopithecus_talapoin Papio_anubis
strepsirhines Eulemur_rubriventer Otolemur_garnettii
Eulemur  Eulemur_mongoz Eulemur_rubriventer
lemuriforms  Varecia_variegata Eulemur_mongoz
lorisiforms  Nycticebus_coucang Otolemur_garnettii
bats  Pteropus_hypomelanus Myotis_velifer 
Pteropus  Pteropus_hypomelanus Pteropus_vampyrus
microbats  Artibeus_jamaicensis Myotis_velifer 
perissodactyls Diceros_bicornis Equus_zebra
Equus  Equus_ferus  Equus_zebra
cetartiodactyls Camelus_ferus Ovis_dalli
artiofabula Pecari_tajacu Balaenoptera_acutorostrata
cetruminants Tursiops_truncatus Ovis_dalli
cetaceans Stenella_attenuata Megaptera_novaeangliae
dolphins Stenella_attenuata Tursiops_truncatus
whales Balaenoptera_acutorostrata Megaptera_novaeangliae
ruminants Giraffa_camelopardalis Ovis_dalli
bovids+cervids Bos_gaurus Alces_alces
bovids Bos_gaurus Ovis_dalli 
cervids Alces_alces Cervus_nippon
glires Castor_fiber Oryctolagus_cuniculus
rodents Castor_fiber Thrichomys_apereoides
lagomorphs Lepus_europaeus Oryctolagus_cuniculus
elephants Elephas_maximus Loxodonta_africana
euarchontoglires Eulemur_mongoz Oryctolagus_cuniculus
boreoeutheria Eulemur_mongoz Panthera_leo
laurasiatheria Panthera_leo Crocidura_russula
scrotifera Panthera_leo Myotis_velifer
ferungulata Panthera_leo Camelus_ferus", stringsAsFactors=FALSE)


names(labDat) <- c("clade","tip1","tip2")
labDat
```

```
##               clade                       tip1                       tip2
## 1        monotremes   Ornithorhynchus_anatinus     Tachyglossus_aculeatus
## 2        marsupials             Macropus_rufus       Didelphis_virginiana
## 3      diprotodonts             Macropus_rufus     Phascolarctos_cinereus
## 4    macropodiforms             Macropus_rufus         Bettongia_gaimardi
## 5        placentals               Panthera_leo         Loxodonta_africana
## 6        carnivores               Panthera_leo    Arctocephalus_australis
## 7            canids              Vulpes_vulpes   Nyctereutes_procyonoides
## 8          arctoids             Neovison_vison    Arctocephalus_australis
## 9        musteloids             Neovison_vison          Mephitis_mephitis
## 10           ursids               Ursus_arctos           Ursus_americanus
## 11        pinnipeds    Leptonychotes_weddellii    Arctocephalus_australis
## 12          phocids    Leptonychotes_weddellii             Phoca_vitulina
## 13         otariids        Callorhinus_ursinus    Arctocephalus_australis
## 14    Arctocephalus    Arctocephalus_australis   Arctocephalus_tropicalis
## 15         Mirounga           Mirounga_leonina    Mirounga_angustirostris
## 16         primates           Gorilla_beringei             Eulemur_mongoz
## 17      anthropoids       Miopithecus_talapoin          Alouatta_palliata
## 18      catarrhines       Miopithecus_talapoin           Gorilla_beringei
## 19  cercopithecoids       Miopithecus_talapoin               Papio_anubis
## 20    strepsirhines        Eulemur_rubriventer         Otolemur_garnettii
## 21          Eulemur             Eulemur_mongoz        Eulemur_rubriventer
## 22      lemuriforms          Varecia_variegata             Eulemur_mongoz
## 23      lorisiforms         Nycticebus_coucang         Otolemur_garnettii
## 24             bats       Pteropus_hypomelanus             Myotis_velifer
## 25         Pteropus       Pteropus_hypomelanus          Pteropus_vampyrus
## 26        microbats       Artibeus_jamaicensis             Myotis_velifer
## 27   perissodactyls           Diceros_bicornis                Equus_zebra
## 28            Equus                Equus_ferus                Equus_zebra
## 29  cetartiodactyls              Camelus_ferus                 Ovis_dalli
## 30      artiofabula              Pecari_tajacu Balaenoptera_acutorostrata
## 31     cetruminants         Tursiops_truncatus                 Ovis_dalli
## 32        cetaceans         Stenella_attenuata     Megaptera_novaeangliae
## 33         dolphins         Stenella_attenuata         Tursiops_truncatus
## 34           whales Balaenoptera_acutorostrata     Megaptera_novaeangliae
## 35        ruminants     Giraffa_camelopardalis                 Ovis_dalli
## 36   bovids+cervids                 Bos_gaurus                Alces_alces
## 37           bovids                 Bos_gaurus                 Ovis_dalli
## 38          cervids                Alces_alces              Cervus_nippon
## 39           glires               Castor_fiber      Oryctolagus_cuniculus
## 40          rodents               Castor_fiber      Thrichomys_apereoides
## 41       lagomorphs            Lepus_europaeus      Oryctolagus_cuniculus
## 42        elephants            Elephas_maximus         Loxodonta_africana
## 43 euarchontoglires             Eulemur_mongoz      Oryctolagus_cuniculus
## 44    boreoeutheria             Eulemur_mongoz               Panthera_leo
## 45   laurasiatheria               Panthera_leo          Crocidura_russula
## 46       scrotifera               Panthera_leo             Myotis_velifer
## 47      ferungulata               Panthera_leo              Camelus_ferus
```

```
for (i in 1:nrow(labDat)){
    idx <- getMRCA(phy=consTreeLab, tip=c(labDat$tip1[i],labDat$tip2[i])) - length(consTreeLab$tip.label)
    consTreeLab$node.label[idx] <- labDat$clade[i]
}
##consTreeLab$node.label
```

Run univariate KSI tests.

```
Fat <- milk$Fat
Fat <- setNames(Fat, milk$phyName) ## needed for ksi()
ksiFat <- ksi(consTreeLab, dat=Fat)
```

```
## Loading required package: Peacock.test
```

```
## Using 'ks' tests
## depth = 1...best node: 13 -- pinnipeds
## depth = 2...best node: 59 -- cetaceans
## depth = 3...best node: 30 -- perissodactyls
## depth = 4...best node: 72 -- primates
## depth = 5...best node: 85 -- Eulemur
## depth = 6...best node: 111 -- nd116
## depth = 7...best node: 7 -- ferungulata
## depth = 8...best node: 66 -- Pteropus
## depth = 9...best node: 85 -- lorisiforms
## depth = 10...best node: 15 -- nd17
```

```
Prot <- milk$Protein
Prot <- setNames(Prot, milk$phyName)
ksiProt <- ksi(consTreeLab, dat=Prot)
```

```
## Using 'ks' tests
## depth = 1...best node: 75 -- primates
## depth = 2...best node: 31 -- perissodactyls
## depth = 3...best node: 69 -- Pteropus
## depth = 4...best node: 39 -- ruminants
## depth = 5...best node: 85 -- Eulemur
## depth = 6...best node: 73 -- catarrhines
## depth = 7...best node: 72 -- anthropoids
## depth = 8...best node: 59 -- whales
## depth = 9...best node: 16 -- Arctocephalus
## depth = 10...best node: 47 -- nd50
```

```
Sug <- milk$Sugar[is.na(milk$Sugar)==FALSE]
Sug <- setNames(Sug, milk$phyName[is.na(milk$Sugar)==FALSE])
ksiSug <- ksi(consTreeLab, dat=Sug)
```

```
## Using 'ks' tests
## depth = 1...best node: 62 -- primates
## depth = 2...best node: 13 -- pinnipeds
## depth = 3...best node: 100 -- nd116
## depth = 4...best node: 23 -- perissodactyls
## depth = 5...best node: 55 -- Pteropus
## depth = 6...best node: 47 -- cetaceans
## depth = 7...best node: 70 -- Eulemur
## depth = 8...best node: 57 -- euarchontoglires
## depth = 9...best node: 71 -- lorisiforms
## depth = 10...best node: 8 -- nd8
```

With the tweaked ksi package, 2- and 3-variate tests are also possible.
The 3-variate test is *much* slower.

```
nomiss <- subset(milk, is.na(milk$Sugar)==FALSE )
nrow(milk) - nrow(nomiss)  ## loss of 15 species if sugar included, using raw data not logit-transformed
```

```
## [1] 15
```

```
system.time( ksiFatProt <- ksi(consTreeLab, dat=milk[,c("Fat","Protein")], test = "p23") )
```

```
## Using 'p23' tests
## depth = 1...best node: 13 -- pinnipeds
## depth = 2...best node: 74 -- primates
## depth = 3...best node: 30 -- perissodactyls
## depth = 4...best node: 58 -- cetaceans
## depth = 5...best node: 67 -- Pteropus
## depth = 6...best node: 35 -- cetartiodactyls
## depth = 7...best node: 71 -- catarrhines
## depth = 8...best node: 109 -- nd116
## depth = 9...best node: 13 -- otariids
## depth = 10...best node: 81 -- Eulemur
```

```
##    user  system elapsed 
##  11.409   0.000  11.413
```

```
system.time( ksiFatSug <- ksi(consTreeLab, dat=nomiss[,c("Fat","Sugar")], test = "p23") )
```

```
## Using 'p23' tests
## depth = 1...best node: 62 -- primates
## depth = 2...best node: 13 -- pinnipeds
## depth = 3...best node: 23 -- perissodactyls
## depth = 4...best node: 30 -- bovids+cervids
## depth = 5...best node: 96 -- nd114
## depth = 6...best node: 54 -- Pteropus
## depth = 7...best node: 29 -- cetruminants
## depth = 8...best node: 68 -- lemuriforms
## depth = 9...best node: 48 -- bats
## depth = 10...best node: 4 -- boreoeutheria
```

```
##    user  system elapsed 
##   7.171   0.000   7.174
```

```
system.time( ksiProtSug <- ksi(consTreeLab, dat=nomiss[,c("Protein","Sugar")], test = "p23") )
```

```
## Using 'p23' tests
## depth = 1...best node: 62 -- primates
## depth = 2...best node: 13 -- pinnipeds
## depth = 3...best node: 96 -- marsupials
## depth = 4...best node: 23 -- perissodactyls
## depth = 5...best node: 55 -- Pteropus
## depth = 6...best node: 30 -- bovids+cervids
## depth = 7...best node: 58 -- anthropoids
## depth = 8...best node: 29 -- cetruminants
## depth = 9...best node: 34 -- nd49
## depth = 10...best node: 93 -- nd116
```

```
##    user  system elapsed 
##   7.215   0.000   7.218
```

```
system.time( ksiFatProtSug <- ksi(consTreeLab, dat=nomiss[,c("Fat","Protein","Sugar")], test = "p23") )
```

```
## Using 'p23' tests
## depth = 1...best node: 62 -- primates
## depth = 2...best node: 32 -- bovids+cervids
## depth = 3...best node: 12 -- nd12
## depth = 4...best node: 23 -- perissodactyls
## depth = 5...best node: 94 -- marsupials
## depth = 6...best node: 54 -- Pteropus
## depth = 7...best node: 59 -- catarrhines
## depth = 8...best node: 8 -- nd8
## depth = 9...best node: 28 -- cetruminants
## depth = 10...best node: 47 -- bats
```

```
##    user  system elapsed 
## 743.208   0.000 743.383
```

Decline in KSI stat for top 10 clades.

```
ksiStats <- data.frame(rank=1:10,
                       Fat=summary(ksiFat)$statistic.rel,
                       Protein=summary(ksiProt)$statistic.rel,
                       Sugar=summary(ksiSug)$statistic.rel,
                       FatProt=summary(ksiFatProt)$statistic.rel,
                       FatSug=summary(ksiFatSug)$statistic.rel,
                       ProtSug=summary(ksiProtSug)$statistic.rel,
                       FatProtSug=summary(ksiFatProtSug)$statistic.rel)
ksim <- melt(ksiStats, id.vars="rank")
gksim <- ggplot(ksim, aes(x=rank, y=value, color=variable)) + geom_line(lwd=2) + theme_classic() + xlab("clade rank") + ylab("K-S relative importance")
```

```
gksim
```

```
ggsave(gksim, filename="output/ksim.pdf", height=6, width=9) ## if desired as figure
```

Summary table of KSI tests showing top 5 ranked clades only.  
Order is Fat, Protein, Sugar, Fat-Protein, Fat-Sugar, Protein-Sugar, Fat-Protein-Sugar.

```
ksiSumAll <- rbind(summary(ksiFat), summary(ksiProt), summary(ksiSug),
                   summary(ksiFatProt), summary(ksiFatSug),  summary(ksiProtSug),
                   summary(ksiFatProtSug) )
## ksiSumAll
ksiSumAll[,3] <- round(ksiSumAll[,3],3)
ksiSumAll[,4] <- round(ksiSumAll[,4],3)
knitr::kable(ksiSumAll[ksiSumAll$rank < 6,], col.names=c("node","rank","KSI","KSI/max","nodesets"), row.names=FALSE)
```

| node | rank | KSI | KSI/max | nodesets |
| --- | --- | --- | --- | --- |
| pinnipeds | 1 | 3.268 | 1.000 | pinnipeds; nd12; arctoids; nd10; carnivores; phocids |
| cetaceans | 2 | 2.221 | 0.680 | cetaceans; whales |
| perissodactyls | 3 | 2.160 | 0.661 | perissodactyls |
| primates | 4 | 2.272 | 0.695 | primates; anthropoids; catarrhines |
| Eulemur | 5 | 1.677 | 0.513 | Eulemur |
| primates | 1 | 3.150 | 1.000 | primates; anthropoids; catarrhines; cercopithecoids |
| perissodactyls | 2 | 2.186 | 0.694 | perissodactyls; Equus; nd33 |
| Pteropus | 3 | 2.178 | 0.691 | Pteropus; bats; nd71 |
| ruminants | 4 | 1.906 | 0.605 | ruminants; bovids+cervids; bovids |
| Eulemur | 5 | 1.565 | 0.497 | Eulemur |
| primates | 1 | 3.301 | 1.000 | primates; anthropoids |
| pinnipeds | 2 | 2.700 | 0.818 | pinnipeds; nd12; phocids |
| nd116 | 3 | 2.169 | 0.657 | nd116; diprotodonts; nd114; nd113; marsupials; nd117 |
| perissodactyls | 4 | 1.926 | 0.584 | perissodactyls; Equus |
| Pteropus | 5 | 1.947 | 0.590 | Pteropus; bats; nd71 |
| pinnipeds | 1 | 3.396 | 1.000 | pinnipeds; nd12; arctoids; nd10 |
| primates | 2 | 3.056 | 0.900 | primates; anthropoids; catarrhines; cercopithecoids |
| perissodactyls | 3 | 2.480 | 0.730 | perissodactyls |
| cetaceans | 4 | 2.237 | 0.659 | cetaceans |
| Pteropus | 5 | 2.162 | 0.637 | Pteropus; bats |
| primates | 1 | 3.433 | 1.000 | primates; anthropoids |
| pinnipeds | 2 | 2.700 | 0.786 | pinnipeds; nd12 |
| perissodactyls | 3 | 2.497 | 0.727 | perissodactyls |
| bovids+cervids | 4 | 2.390 | 0.696 | bovids+cervids |
| nd114 | 5 | 2.141 | 0.624 | nd114; nd113; marsupials; diprotodonts; nd116 |
| primates | 1 | 3.653 | 1.000 | primates; anthropoids |
| pinnipeds | 2 | 2.700 | 0.739 | pinnipeds; nd12 |
| marsupials | 3 | 2.395 | 0.656 | marsupials; nd113; nd114 |
| perissodactyls | 4 | 2.276 | 0.623 | perissodactyls |
| Pteropus | 5 | 2.147 | 0.588 | Pteropus; nd71 |
| primates | 1 | 3.697 | 1.000 | primates; anthropoids |
| bovids+cervids | 2 | 2.846 | 0.770 | bovids+cervids |
| nd12 | 3 | 2.698 | 0.730 | nd12; pinnipeds |
| perissodactyls | 4 | 2.447 | 0.662 | perissodactyls |
| marsupials | 5 | 2.292 | 0.620 | marsupials; nd113; nd114 |

```
cat( knitr::kable(ksiSumAll[ksiSumAll$rank < 6,], col.names=c("node","rank","KSI","KSI/max","nodesets"), row.names=FALSE, format="latex", linesep="", booktabs=TRUE),  file="output/ksiTable.tex") # basis for manuscript table
```

# Selection Regimes

Use the logit-transformed milk composition and transpose for PhylogeneticEM.

```
data <- milk[,c("itFat", "itProtein", "itSugar")]
names(data) <- gsub("it", "", names(data))
tdat <- t(data)
data <- tdat
data[,1:3]
```

```
##         Bos_gaurus Capra_ibex Connochaetes_taurinus
## Fat      -2.586689  -1.955085             -2.512306
## Protein  -2.699549  -2.806015             -3.152319
## Sugar    -2.903111         NA             -2.883007
```

Re-order columns to match phylogeny tips. Avoids later warning from `PhyloEM()`.

```
data <- data[, match(consTree$tip.label, colnames(data)) ]
table( colnames(data) == consTree$tip.label ) ## all match (TRUE)
```

```
## 
## TRUE 
##  124
```

Run `PhyloEM()` scalar OU model. Parallel just makes if faster.

```
system.time( res <- PhyloEM(phylo = consTree,
               Y_data = data,
               process = "scOU",
               random.root = TRUE,
               stationary.root = TRUE,
               parallel_alpha = TRUE,
               Ncores = 4,
               K_max = 12) ) # don't do more than approx. sqrt(Ntaxa)
```

```
## There are some equivalent solutions to the set of shifts selected by the BGHlsq method.
```

```
## There are some equivalent solutions to the set of shifts selected by the BGHml method.
```

```
## There are some equivalent solutions to the set of shifts selected by the BGHlsqraw method.
```

```
## There are some equivalent solutions to the set of shifts selected by the BGHmlraw method.
```

```
##    user  system elapsed 
##   0.326   0.103  38.973
```

```
res
```

```
## Result of the PhyloEM algorithm.
## Selected parameters by the default method:
```

```
## Warning in params_process.PhyloEM(x): There are several equivalent
## solutions for this shift position.
```

```
## 
## 3 dimensional scOU process with a random stationary root.
## 
## Root expectations:
## [1] -1.991134 -2.494678 -3.288971
## 
## Root variance:
##            [,1]        [,2]        [,3]
## [1,]  0.4658784  0.14120268 -0.11691343
## [2,]  0.1412027  0.13802519 -0.05070818
## [3,] -0.1169134 -0.05070818  0.13826878
## 
## Process variance:
##             [,1]         [,2]         [,3]
## [1,]  0.06813359  0.020650550 -0.017098306
## [2,]  0.02065055  0.020185849 -0.007415949
## [3,] -0.01709831 -0.007415949  0.020221474
## 
## Process selection strength:
##            [,1]       [,2]       [,3]
## [1,] 0.07312379 0.00000000 0.00000000
## [2,] 0.00000000 0.07312379 0.00000000
## [3,] 0.00000000 0.00000000 0.07312379
## 
## Process root optimal values:
## [1] -1.991134 -2.494678 -3.288971
## 
## Shifts positions on branches: 144, 221, 53, 12, 35, 13 
## Shifts values:
##             144         221         53          12          35         13
## [1,] -1.4241111 -0.62101372 -2.4147056  2.38757237 -0.60824360 -0.6764136
## [2,] -1.2267008  0.04596896 -1.2235369  0.04933793  0.05099087  0.3373979
## [3,]  0.7298873  0.80659885  0.5493349 -1.57632900 -9.29548028 -2.1666610
## 
## 
## 
## See help to see all plotting and handling functions.
```

Inspect the best K more.
How confident can we be that K=6 is best? K=7 looks OK, too.

```
pdf(file="output/PhyloEMselectK.pdf", height=7, width=7)
plot(res$alpha_max$results_summary$crit_BGHml ~ res$alpha_max$results_summary$K_try ,
     type='b', pch=19, xlab="K (number of clade shifts)", ylab="criterion", main="clade shift selection criteria")
graphics.off()
```

K=6 looks like it's best, but multiple orders to shifts (see warning).
These are root values on transformed scale for fat, protein, and sugar.

```
params_process(res, K = 6)$root.state$exp.root
```

```
## Warning in params_process.PhyloEM(res, K = 6): There are several equivalent
## solutions for this shift position.
```

```
## [1] -1.991134 -2.494678 -3.288971
```

Here they are as raw percentages again.

```
100 * boot::inv.logit(params_process(res, K = 6)$root.state$exp.root)
```

```
## Warning in params_process.PhyloEM(res, K = 6): There are several equivalent
## solutions for this shift position.
```

```
## [1] 12.013689  7.623208  3.595150
```

Process VCV, R in the manuscript notation.
R is sometimes called the “rate matrix”

```
params_process(res, K = 6)$variance
```

```
## Warning in params_process.PhyloEM(res, K = 6): There are several equivalent
## solutions for this shift position.
```

```
## 3 x 3 Matrix of class "dpoMatrix"
##             [,1]         [,2]         [,3]
## [1,]  0.06813359  0.020650550 -0.017098306
## [2,]  0.02065055  0.020185849 -0.007415949
## [3,] -0.01709831 -0.007415949  0.020221474
```

A = selection strength - diagonal matrix with alpha
`t_1/2 = ln(2)/alpha` for phylogenetic half-life

```
params_process(res, K = 6)$selection.strength
```

```
## Warning in params_process.PhyloEM(res, K = 6): There are several equivalent
## solutions for this shift position.
```

```
##            [,1]       [,2]       [,3]
## [1,] 0.07312379 0.00000000 0.00000000
## [2,] 0.00000000 0.07312379 0.00000000
## [3,] 0.00000000 0.00000000 0.07312379
```

```
log(2) / params_process(res, K = 6)$selection.strength[1,1] ## weak!
```

```
## Warning in params_process.PhyloEM(res, K = 6): There are several equivalent
## solutions for this shift position.
```

```
## [1] 9.479093
```

The half-life is 9.5x the tree height, heuristically meaning it would take
this long for a species to move halfway to their optimum.
That's 9.5x the 218 Ma for the mammal MRCA.

```
max(phytools::nodeHeights(consTree)[,2])
```

```
## [1] 217.84
```

That's very weak selection overall
BUT…. this is NOT about the clade shifts.
Those are treated as instantaneous.

Matrix of 3 traits x K shifts.

```
params_process(res, K = 6)$shifts$values
```

```
## Warning in params_process.PhyloEM(res, K = 6): There are several equivalent
## solutions for this shift position.
```

```
##            [,1]        [,2]       [,3]        [,4]        [,5]       [,6]
## [1,] -1.4241111 -0.62101372 -2.4147056  2.38757237 -0.60824360 -0.6764136
## [2,] -1.2267008  0.04596896 -1.2235369  0.04933793  0.05099087  0.3373979
## [3,]  0.7298873  0.80659885  0.5493349 -1.57632900 -9.29548028 -2.1666610
```

Add these shifts vs. the root to get the new clade-optima.

```
apply(params_process(res, K = 6)$shifts$values,2, function(z) 100 * boot::inv.logit(z + params_process(res, K = 6)$root.state$exp.root) )
```

```
## Warning in params_process.PhyloEM(res, K = 6): There are several equivalent
## solutions for this shift position.

## Warning in params_process.PhyloEM(res, K = 6): There are several equivalent
## solutions for this shift position.

## Warning in params_process.PhyloEM(res, K = 6): There are several equivalent
## solutions for this shift position.

## Warning in params_process.PhyloEM(res, K = 6): There are several equivalent
## solutions for this shift position.

## Warning in params_process.PhyloEM(res, K = 6): There are several equivalent
## solutions for this shift position.

## Warning in params_process.PhyloEM(res, K = 6): There are several equivalent
## solutions for this shift position.

## Warning in params_process.PhyloEM(res, K = 6): There are several equivalent
## solutions for this shift position.
```

```
##          [,1]     [,2]     [,3]       [,4]         [,5]       [,6]
## [1,] 3.182239 6.836066 1.205866 59.7831527 6.9178457461  6.4915645
## [2,] 2.362874 7.953297 2.370184  7.9779956 7.9901391199 10.3652840
## [3,] 7.181860 7.710324 6.067464  0.7650534 0.0003424844  0.4254002
```

Get a classification of the tips from the edges to identify taxa
within the clades. Do it for each K.

```
edgetipl <- enumerate_tips_under_edges(consTree)
shiftl <- rep(NA, 10); class(shiftl) <- "list"
for (i in 1:length(shiftl)){
    tipl <- lapply(1:length(params_process(res, K = i)$shifts$edges),
                   function(z) { edgetipl[[params_process(res, K = i)$shifts$edges[z]]] } )
    tiplc <- lapply(tipl, function(z) {
        consTree$tip.label[z]
    })
    shiftl[[i]] <- tiplc
}
```

```
## Warning in params_process.PhyloEM(res, K = i): There are several equivalent
## solutions for this shift position.

## Warning in params_process.PhyloEM(res, K = i): There are several equivalent
## solutions for this shift position.

## Warning in params_process.PhyloEM(res, K = i): There are several equivalent
## solutions for this shift position.

## Warning in params_process.PhyloEM(res, K = i): There are several equivalent
## solutions for this shift position.

## Warning in params_process.PhyloEM(res, K = i): There are several equivalent
## solutions for this shift position.

## Warning in params_process.PhyloEM(res, K = i): There are several equivalent
## solutions for this shift position.

## Warning in params_process.PhyloEM(res, K = i): There are several equivalent
## solutions for this shift position.

## Warning in params_process.PhyloEM(res, K = i): There are several equivalent
## solutions for this shift position.

## Warning in params_process.PhyloEM(res, K = i): There are several equivalent
## solutions for this shift position.

## Warning in params_process.PhyloEM(res, K = i): There are several equivalent
## solutions for this shift position.

## Warning in params_process.PhyloEM(res, K = i): There are several equivalent
## solutions for this shift position.

## Warning in params_process.PhyloEM(res, K = i): There are several equivalent
## solutions for this shift position.

## Warning in params_process.PhyloEM(res, K = i): There are several equivalent
## solutions for this shift position.

## Warning in params_process.PhyloEM(res, K = i): There are several equivalent
## solutions for this shift position.

## Warning in params_process.PhyloEM(res, K = i): There are several equivalent
## solutions for this shift position.

## Warning in params_process.PhyloEM(res, K = i): There are several equivalent
## solutions for this shift position.

## Warning in params_process.PhyloEM(res, K = i): There are several equivalent
## solutions for this shift position.

## Warning in params_process.PhyloEM(res, K = i): There are several equivalent
## solutions for this shift position.

## Warning in params_process.PhyloEM(res, K = i): There are several equivalent
## solutions for this shift position.

## Warning in params_process.PhyloEM(res, K = i): There are several equivalent
## solutions for this shift position.

## Warning in params_process.PhyloEM(res, K = i): There are several equivalent
## solutions for this shift position.

## Warning in params_process.PhyloEM(res, K = i): There are several equivalent
## solutions for this shift position.

## Warning in params_process.PhyloEM(res, K = i): There are several equivalent
## solutions for this shift position.

## Warning in params_process.PhyloEM(res, K = i): There are several equivalent
## solutions for this shift position.

## Warning in params_process.PhyloEM(res, K = i): There are several equivalent
## solutions for this shift position.

## Warning in params_process.PhyloEM(res, K = i): There are several equivalent
## solutions for this shift position.

## Warning in params_process.PhyloEM(res, K = i): There are several equivalent
## solutions for this shift position.

## Warning in params_process.PhyloEM(res, K = i): There are several equivalent
## solutions for this shift position.

## Warning in params_process.PhyloEM(res, K = i): There are several equivalent
## solutions for this shift position.

## Warning in params_process.PhyloEM(res, K = i): There are several equivalent
## solutions for this shift position.

## Warning in params_process.PhyloEM(res, K = i): There are several equivalent
## solutions for this shift position.

## Warning in params_process.PhyloEM(res, K = i): There are several equivalent
## solutions for this shift position.

## Warning in params_process.PhyloEM(res, K = i): There are several equivalent
## solutions for this shift position.

## Warning in params_process.PhyloEM(res, K = i): There are several equivalent
## solutions for this shift position.

## Warning in params_process.PhyloEM(res, K = i): There are several equivalent
## solutions for this shift position.

## Warning in params_process.PhyloEM(res, K = i): There are several equivalent
## solutions for this shift position.

## Warning in params_process.PhyloEM(res, K = i): There are several equivalent
## solutions for this shift position.

## Warning in params_process.PhyloEM(res, K = i): There are several equivalent
## solutions for this shift position.

## Warning in params_process.PhyloEM(res, K = i): There are several equivalent
## solutions for this shift position.

## Warning in params_process.PhyloEM(res, K = i): There are several equivalent
## solutions for this shift position.

## Warning in params_process.PhyloEM(res, K = i): There are several equivalent
## solutions for this shift position.

## Warning in params_process.PhyloEM(res, K = i): There are several equivalent
## solutions for this shift position.

## Warning in params_process.PhyloEM(res, K = i): There are several equivalent
## solutions for this shift position.

## Warning in params_process.PhyloEM(res, K = i): There are several equivalent
## solutions for this shift position.

## Warning in params_process.PhyloEM(res, K = i): There are several equivalent
## solutions for this shift position.

## Warning in params_process.PhyloEM(res, K = i): There are several equivalent
## solutions for this shift position.

## Warning in params_process.PhyloEM(res, K = i): There are several equivalent
## solutions for this shift position.

## Warning in params_process.PhyloEM(res, K = i): There are several equivalent
## solutions for this shift position.

## Warning in params_process.PhyloEM(res, K = i): There are several equivalent
## solutions for this shift position.

## Warning in params_process.PhyloEM(res, K = i): There are several equivalent
## solutions for this shift position.

## Warning in params_process.PhyloEM(res, K = i): There are several equivalent
## solutions for this shift position.
```

```
names(shiftl) <- paste("K", 1:length(shiftl), sep="=")
shiftl
```

```
## $`K=1`
## $`K=1`[[1]]
## [1] "Mirounga_angustirostris" "Mirounga_leonina"       
## 
## 
## $`K=2`
## $`K=2`[[1]]
## [1] "Mirounga_angustirostris" "Mirounga_leonina"       
## 
## $`K=2`[[2]]
##  [1] "Arctocephalus_australis"  "Neophoca_cinerea"        
##  [3] "Arctocephalus_gazella"    "Arctocephalus_pusillus"  
##  [5] "Arctocephalus_tropicalis" "Zalophus_californianus"  
##  [7] "Callorhinus_ursinus"      "Halichoerus_grypus"      
##  [9] "Phoca_vitulina"           "Pagophilus_groenlandicus"
## [11] "Cystophora_cristata"      "Mirounga_angustirostris" 
## [13] "Mirounga_leonina"         "Leptonychotes_weddellii" 
## 
## 
## $`K=3`
## $`K=3`[[1]]
## [1] "Mirounga_angustirostris" "Mirounga_leonina"       
## 
## $`K=3`[[2]]
## [1] "Arctocephalus_australis"  "Neophoca_cinerea"        
## [3] "Arctocephalus_gazella"    "Arctocephalus_pusillus"  
## [5] "Arctocephalus_tropicalis" "Zalophus_californianus"  
## [7] "Callorhinus_ursinus"     
## 
## $`K=3`[[3]]
## [1] "Stenella_attenuata"
## 
## 
## $`K=4`
## $`K=4`[[1]]
## [1] "Mirounga_angustirostris" "Mirounga_leonina"       
## 
## $`K=4`[[2]]
## [1] "Arctocephalus_australis"  "Neophoca_cinerea"        
## [3] "Arctocephalus_gazella"    "Arctocephalus_pusillus"  
## [5] "Arctocephalus_tropicalis" "Zalophus_californianus"  
## [7] "Callorhinus_ursinus"     
## 
## $`K=4`[[3]]
## [1] "Setonix_brachyurus"
## 
## $`K=4`[[4]]
## [1] "Stenella_attenuata"
## 
## 
## $`K=5`
## $`K=5`[[1]]
## [1] "Mirounga_angustirostris" "Mirounga_leonina"       
## 
## $`K=5`[[2]]
##  [1] "Arctocephalus_australis"  "Neophoca_cinerea"        
##  [3] "Arctocephalus_gazella"    "Arctocephalus_pusillus"  
##  [5] "Arctocephalus_tropicalis" "Zalophus_californianus"  
##  [7] "Callorhinus_ursinus"      "Halichoerus_grypus"      
##  [9] "Phoca_vitulina"           "Pagophilus_groenlandicus"
## [11] "Cystophora_cristata"      "Mirounga_angustirostris" 
## [13] "Mirounga_leonina"         "Leptonychotes_weddellii" 
## 
## $`K=5`[[3]]
## [1] "Arctocephalus_australis"  "Neophoca_cinerea"        
## [3] "Arctocephalus_gazella"    "Arctocephalus_pusillus"  
## [5] "Arctocephalus_tropicalis" "Zalophus_californianus"  
## [7] "Callorhinus_ursinus"     
## 
## $`K=5`[[4]]
## [1] "Stenella_attenuata"
## 
## $`K=5`[[5]]
## [1] "Setonix_brachyurus"
## 
## 
## $`K=6`
## $`K=6`[[1]]
##  [1] "Macaca_fuscata"          "Macaca_mulatta"         
##  [3] "Macaca_fascicularis"     "Papio_anubis"           
##  [5] "Miopithecus_talapoin"    "Chlorocebus_pygerythrus"
##  [7] "Gorilla_beringei"        "Callithrix_jacchus"     
##  [9] "Leontopithecus_rosalia"  "Saimiri_boliviensis"    
## [11] "Alouatta_palliata"       "Alouatta_seniculus"     
## [13] "Eulemur_mongoz"          "Eulemur_fulvus"         
## [15] "Eulemur_macaco"          "Eulemur_rubriventer"    
## [17] "Varecia_variegata"       "Otolemur_crassicaudatus"
## [19] "Otolemur_garnettii"      "Nycticebus_coucang"     
## 
## $`K=6`[[2]]
##  [1] "Macropus_rufogriseus"     "Macropus_eugenii"        
##  [3] "Macropus_rufus"           "Setonix_brachyurus"      
##  [5] "Potorous_tridactylus"     "Bettongia_gaimardi"      
##  [7] "Pseudocheirus_peregrinus" "Trichosurus_vulpecula"   
##  [9] "Phascolarctos_cinereus"   "Isoodon_macrourus"       
## [11] "Dasyurus_viverrinus"      "Didelphis_virginiana"    
## 
## $`K=6`[[3]]
## [1] "Equus_africanus"    "Equus_zebra"        "Equus_quagga"      
## [4] "Equus_ferus"        "Tapirus_bairdii"    "Tapirus_terrestris"
## [7] "Diceros_bicornis"  
## 
## $`K=6`[[4]]
##  [1] "Arctocephalus_australis"  "Neophoca_cinerea"        
##  [3] "Arctocephalus_gazella"    "Arctocephalus_pusillus"  
##  [5] "Arctocephalus_tropicalis" "Zalophus_californianus"  
##  [7] "Callorhinus_ursinus"      "Halichoerus_grypus"      
##  [9] "Phoca_vitulina"           "Pagophilus_groenlandicus"
## [11] "Cystophora_cristata"      "Mirounga_angustirostris" 
## [13] "Mirounga_leonina"         "Leptonychotes_weddellii" 
## 
## $`K=6`[[5]]
## [1] "Mirounga_angustirostris" "Mirounga_leonina"       
## 
## $`K=6`[[6]]
## [1] "Arctocephalus_australis"  "Neophoca_cinerea"        
## [3] "Arctocephalus_gazella"    "Arctocephalus_pusillus"  
## [5] "Arctocephalus_tropicalis" "Zalophus_californianus"  
## [7] "Callorhinus_ursinus"     
## 
## 
## $`K=7`
## $`K=7`[[1]]
##  [1] "Macaca_fuscata"          "Macaca_mulatta"         
##  [3] "Macaca_fascicularis"     "Papio_anubis"           
##  [5] "Miopithecus_talapoin"    "Chlorocebus_pygerythrus"
##  [7] "Gorilla_beringei"        "Callithrix_jacchus"     
##  [9] "Leontopithecus_rosalia"  "Saimiri_boliviensis"    
## [11] "Alouatta_palliata"       "Alouatta_seniculus"     
## [13] "Eulemur_mongoz"          "Eulemur_fulvus"         
## [15] "Eulemur_macaco"          "Eulemur_rubriventer"    
## [17] "Varecia_variegata"       "Otolemur_crassicaudatus"
## [19] "Otolemur_garnettii"      "Nycticebus_coucang"     
## 
## $`K=7`[[2]]
## [1] "Equus_africanus"    "Equus_zebra"        "Equus_quagga"      
## [4] "Equus_ferus"        "Tapirus_bairdii"    "Tapirus_terrestris"
## [7] "Diceros_bicornis"  
## 
## $`K=7`[[3]]
## [1] "Macropus_rufogriseus"     "Macropus_eugenii"        
## [3] "Macropus_rufus"           "Setonix_brachyurus"      
## [5] "Potorous_tridactylus"     "Bettongia_gaimardi"      
## [7] "Pseudocheirus_peregrinus" "Trichosurus_vulpecula"   
## 
## $`K=7`[[4]]
##  [1] "Arctocephalus_australis"  "Neophoca_cinerea"        
##  [3] "Arctocephalus_gazella"    "Arctocephalus_pusillus"  
##  [5] "Arctocephalus_tropicalis" "Zalophus_californianus"  
##  [7] "Callorhinus_ursinus"      "Halichoerus_grypus"      
##  [9] "Phoca_vitulina"           "Pagophilus_groenlandicus"
## [11] "Cystophora_cristata"      "Mirounga_angustirostris" 
## [13] "Mirounga_leonina"         "Leptonychotes_weddellii" 
## 
## $`K=7`[[5]]
## [1] "Mirounga_angustirostris" "Mirounga_leonina"       
## 
## $`K=7`[[6]]
## [1] "Arctocephalus_australis"  "Neophoca_cinerea"        
## [3] "Arctocephalus_gazella"    "Arctocephalus_pusillus"  
## [5] "Arctocephalus_tropicalis" "Zalophus_californianus"  
## [7] "Callorhinus_ursinus"     
## 
## $`K=7`[[7]]
## [1] "Setonix_brachyurus"
## 
## 
## $`K=8`
## $`K=8`[[1]]
##  [1] "Macaca_fuscata"          "Macaca_mulatta"         
##  [3] "Macaca_fascicularis"     "Papio_anubis"           
##  [5] "Miopithecus_talapoin"    "Chlorocebus_pygerythrus"
##  [7] "Gorilla_beringei"        "Callithrix_jacchus"     
##  [9] "Leontopithecus_rosalia"  "Saimiri_boliviensis"    
## [11] "Alouatta_palliata"       "Alouatta_seniculus"     
## [13] "Eulemur_mongoz"          "Eulemur_fulvus"         
## [15] "Eulemur_macaco"          "Eulemur_rubriventer"    
## [17] "Varecia_variegata"       "Otolemur_crassicaudatus"
## [19] "Otolemur_garnettii"      "Nycticebus_coucang"     
## 
## $`K=8`[[2]]
## [1] "Macropus_rufogriseus"     "Macropus_eugenii"        
## [3] "Macropus_rufus"           "Setonix_brachyurus"      
## [5] "Potorous_tridactylus"     "Bettongia_gaimardi"      
## [7] "Pseudocheirus_peregrinus" "Trichosurus_vulpecula"   
## 
## $`K=8`[[3]]
## [1] "Equus_africanus"    "Equus_zebra"        "Equus_quagga"      
## [4] "Equus_ferus"        "Tapirus_bairdii"    "Tapirus_terrestris"
## [7] "Diceros_bicornis"  
## 
## $`K=8`[[4]]
## [1] "Mirounga_angustirostris" "Mirounga_leonina"       
## 
## $`K=8`[[5]]
##  [1] "Arctocephalus_australis"  "Neophoca_cinerea"        
##  [3] "Arctocephalus_gazella"    "Arctocephalus_pusillus"  
##  [5] "Arctocephalus_tropicalis" "Zalophus_californianus"  
##  [7] "Callorhinus_ursinus"      "Halichoerus_grypus"      
##  [9] "Phoca_vitulina"           "Pagophilus_groenlandicus"
## [11] "Cystophora_cristata"      "Mirounga_angustirostris" 
## [13] "Mirounga_leonina"         "Leptonychotes_weddellii" 
## 
## $`K=8`[[6]]
## [1] "Arctocephalus_australis"  "Neophoca_cinerea"        
## [3] "Arctocephalus_gazella"    "Arctocephalus_pusillus"  
## [5] "Arctocephalus_tropicalis" "Zalophus_californianus"  
## [7] "Callorhinus_ursinus"     
## 
## $`K=8`[[7]]
## [1] "Setonix_brachyurus"
## 
## $`K=8`[[8]]
## [1] "Stenella_attenuata"
## 
## 
## $`K=9`
## $`K=9`[[1]]
##  [1] "Macaca_fuscata"          "Macaca_mulatta"         
##  [3] "Macaca_fascicularis"     "Papio_anubis"           
##  [5] "Miopithecus_talapoin"    "Chlorocebus_pygerythrus"
##  [7] "Gorilla_beringei"        "Callithrix_jacchus"     
##  [9] "Leontopithecus_rosalia"  "Saimiri_boliviensis"    
## [11] "Alouatta_palliata"       "Alouatta_seniculus"     
## [13] "Eulemur_mongoz"          "Eulemur_fulvus"         
## [15] "Eulemur_macaco"          "Eulemur_rubriventer"    
## [17] "Varecia_variegata"       "Otolemur_crassicaudatus"
## [19] "Otolemur_garnettii"      "Nycticebus_coucang"     
## 
## $`K=9`[[2]]
## [1] "Macropus_rufogriseus"     "Macropus_eugenii"        
## [3] "Macropus_rufus"           "Setonix_brachyurus"      
## [5] "Potorous_tridactylus"     "Bettongia_gaimardi"      
## [7] "Pseudocheirus_peregrinus" "Trichosurus_vulpecula"   
## 
## $`K=9`[[3]]
## [1] "Equus_africanus"    "Equus_zebra"        "Equus_quagga"      
## [4] "Equus_ferus"        "Tapirus_bairdii"    "Tapirus_terrestris"
## [7] "Diceros_bicornis"  
## 
## $`K=9`[[4]]
## [1] "Mirounga_angustirostris" "Mirounga_leonina"       
## 
## $`K=9`[[5]]
##  [1] "Arctocephalus_australis"  "Neophoca_cinerea"        
##  [3] "Arctocephalus_gazella"    "Arctocephalus_pusillus"  
##  [5] "Arctocephalus_tropicalis" "Zalophus_californianus"  
##  [7] "Callorhinus_ursinus"      "Halichoerus_grypus"      
##  [9] "Phoca_vitulina"           "Pagophilus_groenlandicus"
## [11] "Cystophora_cristata"      "Mirounga_angustirostris" 
## [13] "Mirounga_leonina"         "Leptonychotes_weddellii" 
## 
## $`K=9`[[6]]
## [1] "Arctocephalus_australis"  "Neophoca_cinerea"        
## [3] "Arctocephalus_gazella"    "Arctocephalus_pusillus"  
## [5] "Arctocephalus_tropicalis" "Zalophus_californianus"  
## [7] "Callorhinus_ursinus"     
## 
## $`K=9`[[7]]
## [1] "Setonix_brachyurus"
## 
## $`K=9`[[8]]
## [1] "Stenella_attenuata"
## 
## $`K=9`[[9]]
## [1] "Cervus_astylodon"
## 
## 
## $`K=10`
## $`K=10`[[1]]
##  [1] "Macaca_fuscata"          "Macaca_mulatta"         
##  [3] "Macaca_fascicularis"     "Papio_anubis"           
##  [5] "Miopithecus_talapoin"    "Chlorocebus_pygerythrus"
##  [7] "Gorilla_beringei"        "Callithrix_jacchus"     
##  [9] "Leontopithecus_rosalia"  "Saimiri_boliviensis"    
## [11] "Alouatta_palliata"       "Alouatta_seniculus"     
## [13] "Eulemur_mongoz"          "Eulemur_fulvus"         
## [15] "Eulemur_macaco"          "Eulemur_rubriventer"    
## [17] "Varecia_variegata"       "Otolemur_crassicaudatus"
## [19] "Otolemur_garnettii"      "Nycticebus_coucang"     
## 
## $`K=10`[[2]]
## [1] "Macropus_rufogriseus"     "Macropus_eugenii"        
## [3] "Macropus_rufus"           "Setonix_brachyurus"      
## [5] "Potorous_tridactylus"     "Bettongia_gaimardi"      
## [7] "Pseudocheirus_peregrinus" "Trichosurus_vulpecula"   
## 
## $`K=10`[[3]]
## [1] "Equus_africanus"    "Equus_zebra"        "Equus_quagga"      
## [4] "Equus_ferus"        "Tapirus_bairdii"    "Tapirus_terrestris"
## [7] "Diceros_bicornis"  
## 
## $`K=10`[[4]]
## [1] "Mirounga_angustirostris" "Mirounga_leonina"       
## 
## $`K=10`[[5]]
##  [1] "Arctocephalus_australis"  "Neophoca_cinerea"        
##  [3] "Arctocephalus_gazella"    "Arctocephalus_pusillus"  
##  [5] "Arctocephalus_tropicalis" "Zalophus_californianus"  
##  [7] "Callorhinus_ursinus"      "Halichoerus_grypus"      
##  [9] "Phoca_vitulina"           "Pagophilus_groenlandicus"
## [11] "Cystophora_cristata"      "Mirounga_angustirostris" 
## [13] "Mirounga_leonina"         "Leptonychotes_weddellii" 
## 
## $`K=10`[[6]]
## [1] "Arctocephalus_australis"  "Neophoca_cinerea"        
## [3] "Arctocephalus_gazella"    "Arctocephalus_pusillus"  
## [5] "Arctocephalus_tropicalis" "Zalophus_californianus"  
## [7] "Callorhinus_ursinus"     
## 
## $`K=10`[[7]]
## [1] "Setonix_brachyurus"
## 
## $`K=10`[[8]]
## [1] "Stenella_attenuata"
## 
## $`K=10`[[9]]
## [1] "Cervus_astylodon"
## 
## $`K=10`[[10]]
## [1] "Neophoca_cinerea"
```

K=1 Mirounga

```
plot(res, params = params_process(res, K = 1), traits=c(1,2))
```

K=2 Mirounga, pinnipeds

```
plot(res, params = params_process(res, K = 2), traits=c(1,2))
```

K=3 Mirounga, otariids, spotted dolphin

```
plot(res, params = params_process(res, K = 3), traits=c(1,2))
```

K=4 Mirounga, otariids, quokka, spotted dolphin

```
plot(res, params = params_process(res, K = 4), traits=c(1,2))
```

K=5 Mirounga, pinnipeds, otariids, spotted dolphin, quokka - but multiple alternatives

```
plot(res, params = params_process(res, K = 5), traits=c(1,2))
```

```
## Warning in params_process.PhyloEM(res, K = 5): There are several equivalent
## solutions for this shift position.
```

K=6 primates, marsupials, perissodactyls, pinnipeds, Mirounga, otariids - BEST, but multiple alternatives

```
plot(res, params = params_process(res, K = 6), traits=c(1,2))
```

```
## Warning in params_process.PhyloEM(res, K = 6): There are several equivalent
## solutions for this shift position.
```

K=7 primates, perissodactyls, diprotodonts, pinnipeds, Mirounga, otariids, quokka - but multiple alternatives

```
plot(res, params = params_process(res, K = 7), traits=c(1,2))
```

```
## Warning in params_process.PhyloEM(res, K = 7): There are several equivalent
## solutions for this shift position.
```

K=8 primates, diprotodonts, perissodactyls, Mirounga, pinnipeds, otariids, quokka, spotted dolphin

```
plot(res, params = params_process(res, K = 8), traits=c(1,2))
```

```
## Warning in params_process.PhyloEM(res, K = 8): There are several equivalent
## solutions for this shift position.
```

What are the alternative clade shifts for K=5 through K=8?

```
params_5 <- params_process(res, K = 5)
```

```
## Warning in params_process.PhyloEM(res, K = 5): There are several equivalent
## solutions for this shift position.
```

```
params_6 <- params_process(res, K = 6)
```

```
## Warning in params_process.PhyloEM(res, K = 6): There are several equivalent
## solutions for this shift position.
```

```
params_7 <- params_process(res, K = 7)
```

```
## Warning in params_process.PhyloEM(res, K = 7): There are several equivalent
## solutions for this shift position.
```

```
params_8 <- params_process(res, K = 8)
```

```
## Warning in params_process.PhyloEM(res, K = 8): There are several equivalent
## solutions for this shift position.
```

It's just how the clade shifts are ordered
within pinnipeds for any of these.

```
plot(equivalent_shifts(consTree, params_5))
```

```
plot(equivalent_shifts(consTree, params_6))
```

```
plot(equivalent_shifts(consTree, params_7))
```

```
plot(equivalent_shifts(consTree, params_8))
```

What are the shift optima when K=6?
Inferred root values, transformed scale.

```
params_process(res, K = 6)$root.state$exp.root
```

```
## Warning in params_process.PhyloEM(res, K = 6): There are several equivalent
## solutions for this shift position.
```

```
## [1] -1.991134 -2.494678 -3.288971
```

edges/branches where shifts were inferred

```
params_process(res, K = 6)$shifts$edges
```

```
## Warning in params_process.PhyloEM(res, K = 6): There are several equivalent
## solutions for this shift position.
```

```
## [1] 144 221  53  12  35  13
```

```
params_process(res, K = 6)$shifts$values
```

```
## Warning in params_process.PhyloEM(res, K = 6): There are several equivalent
## solutions for this shift position.
```

```
##            [,1]        [,2]       [,3]        [,4]        [,5]       [,6]
## [1,] -1.4241111 -0.62101372 -2.4147056  2.38757237 -0.60824360 -0.6764136
## [2,] -1.2267008  0.04596896 -1.2235369  0.04933793  0.05099087  0.3373979
## [3,]  0.7298873  0.80659885  0.5493349 -1.57632900 -9.29548028 -2.1666610
```

```
params_process(res, K = 6)$selection.strength
```

```
## Warning in params_process.PhyloEM(res, K = 6): There are several equivalent
## solutions for this shift position.
```

```
##            [,1]       [,2]       [,3]
## [1,] 0.07312379 0.00000000 0.00000000
## [2,] 0.00000000 0.07312379 0.00000000
## [3,] 0.00000000 0.00000000 0.07312379
```

Recall these are the new optima for those clades

```
matrix(params_process(res, K = 6)$root.state$exp.root, ncol=1, nrow=3)
```

```
## Warning in params_process.PhyloEM(res, K = 6): There are several equivalent
## solutions for this shift position.
```

```
##           [,1]
## [1,] -1.991134
## [2,] -2.494678
## [3,] -3.288971
```

```
params_process(res, K = 6)$shifts$values
```

```
## Warning in params_process.PhyloEM(res, K = 6): There are several equivalent
## solutions for this shift position.
```

```
##            [,1]        [,2]       [,3]        [,4]        [,5]       [,6]
## [1,] -1.4241111 -0.62101372 -2.4147056  2.38757237 -0.60824360 -0.6764136
## [2,] -1.2267008  0.04596896 -1.2235369  0.04933793  0.05099087  0.3373979
## [3,]  0.7298873  0.80659885  0.5493349 -1.57632900 -9.29548028 -2.1666610
```

```
shiftl$`K=6` ## these are the clade members
```

```
## [[1]]
##  [1] "Macaca_fuscata"          "Macaca_mulatta"         
##  [3] "Macaca_fascicularis"     "Papio_anubis"           
##  [5] "Miopithecus_talapoin"    "Chlorocebus_pygerythrus"
##  [7] "Gorilla_beringei"        "Callithrix_jacchus"     
##  [9] "Leontopithecus_rosalia"  "Saimiri_boliviensis"    
## [11] "Alouatta_palliata"       "Alouatta_seniculus"     
## [13] "Eulemur_mongoz"          "Eulemur_fulvus"         
## [15] "Eulemur_macaco"          "Eulemur_rubriventer"    
## [17] "Varecia_variegata"       "Otolemur_crassicaudatus"
## [19] "Otolemur_garnettii"      "Nycticebus_coucang"     
## 
## [[2]]
##  [1] "Macropus_rufogriseus"     "Macropus_eugenii"        
##  [3] "Macropus_rufus"           "Setonix_brachyurus"      
##  [5] "Potorous_tridactylus"     "Bettongia_gaimardi"      
##  [7] "Pseudocheirus_peregrinus" "Trichosurus_vulpecula"   
##  [9] "Phascolarctos_cinereus"   "Isoodon_macrourus"       
## [11] "Dasyurus_viverrinus"      "Didelphis_virginiana"    
## 
## [[3]]
## [1] "Equus_africanus"    "Equus_zebra"        "Equus_quagga"      
## [4] "Equus_ferus"        "Tapirus_bairdii"    "Tapirus_terrestris"
## [7] "Diceros_bicornis"  
## 
## [[4]]
##  [1] "Arctocephalus_australis"  "Neophoca_cinerea"        
##  [3] "Arctocephalus_gazella"    "Arctocephalus_pusillus"  
##  [5] "Arctocephalus_tropicalis" "Zalophus_californianus"  
##  [7] "Callorhinus_ursinus"      "Halichoerus_grypus"      
##  [9] "Phoca_vitulina"           "Pagophilus_groenlandicus"
## [11] "Cystophora_cristata"      "Mirounga_angustirostris" 
## [13] "Mirounga_leonina"         "Leptonychotes_weddellii" 
## 
## [[5]]
## [1] "Mirounga_angustirostris" "Mirounga_leonina"       
## 
## [[6]]
## [1] "Arctocephalus_australis"  "Neophoca_cinerea"        
## [3] "Arctocephalus_gazella"    "Arctocephalus_pusillus"  
## [5] "Arctocephalus_tropicalis" "Zalophus_californianus"  
## [7] "Callorhinus_ursinus"
```

```
apply(params_process(res, K = 6)$shifts$values, 2, function(z) 100 * boot::inv.logit(z + params_process(res, K = 6)$root.state$exp.root) )
```

```
## Warning in params_process.PhyloEM(res, K = 6): There are several equivalent
## solutions for this shift position.

## Warning in params_process.PhyloEM(res, K = 6): There are several equivalent
## solutions for this shift position.

## Warning in params_process.PhyloEM(res, K = 6): There are several equivalent
## solutions for this shift position.

## Warning in params_process.PhyloEM(res, K = 6): There are several equivalent
## solutions for this shift position.

## Warning in params_process.PhyloEM(res, K = 6): There are several equivalent
## solutions for this shift position.

## Warning in params_process.PhyloEM(res, K = 6): There are several equivalent
## solutions for this shift position.

## Warning in params_process.PhyloEM(res, K = 6): There are several equivalent
## solutions for this shift position.
```

```
##          [,1]     [,2]     [,3]       [,4]         [,5]       [,6]
## [1,] 3.182239 6.836066 1.205866 59.7831527 6.9178457461  6.4915645
## [2,] 2.362874 7.953297 2.370184  7.9779956 7.9901391199 10.3652840
## [3,] 7.181860 7.710324 6.067464  0.7650534 0.0003424844  0.4254002
```

The primate, marsupial, perissodactyl, and pinniped values are sensible `[,1:4]`
the Mirougna and otariid `[,5:6]` need to be *added* to the pinniped on the logit scale.

Mirounga prediction

```
100 * boot::inv.logit(params_process(res, K = 6)$root.state$exp.root + params_process(res, K = 6)$shifts$values[,4] + params_process(res, K = 6)$shifts$values[,5])
```

```
## Warning in params_process.PhyloEM(res, K = 6): There are several equivalent
## solutions for this shift position.

## Warning in params_process.PhyloEM(res, K = 6): There are several equivalent
## solutions for this shift position.

## Warning in params_process.PhyloEM(res, K = 6): There are several equivalent
## solutions for this shift position.
```

```
## [1] 4.472456e+01 8.360458e+00 7.080289e-05
```

otariid prediction

```
100 * boot::inv.logit(params_process(res, K = 6)$root.state$exp.root + params_process(res, K = 6)$shifts$values[,4] + params_process(res, K = 6)$shifts$values[,6])
```

```
## Warning in params_process.PhyloEM(res, K = 6): There are several equivalent
## solutions for this shift position.

## Warning in params_process.PhyloEM(res, K = 6): There are several equivalent
## solutions for this shift position.

## Warning in params_process.PhyloEM(res, K = 6): There are several equivalent
## solutions for this shift position.
```

```
## [1] 43.04597271 10.83272345  0.08824189
```

```
shiftTab <- data.frame(apply(params_process(res, K = 6)$shifts$values, 2, function(z) 100 * boot::inv.logit(z + params_process(res, K = 6)$root.state$exp.root) )[,1:4], Mirounga = 100 * boot::inv.logit(params_process(res, K = 6)$root.state$exp.root + params_process(res, K = 6)$shifts$values[,4] + params_process(res, K = 6)$shifts$values[,5]), otariids = 100 * boot::inv.logit(params_process(res, K = 6)$root.state$exp.root + params_process(res, K = 6)$shifts$values[,4] + params_process(res, K = 6)$shifts$values[,6]))
```

```
## Warning in params_process.PhyloEM(res, K = 6): There are several equivalent
## solutions for this shift position.

## Warning in params_process.PhyloEM(res, K = 6): There are several equivalent
## solutions for this shift position.

## Warning in params_process.PhyloEM(res, K = 6): There are several equivalent
## solutions for this shift position.

## Warning in params_process.PhyloEM(res, K = 6): There are several equivalent
## solutions for this shift position.

## Warning in params_process.PhyloEM(res, K = 6): There are several equivalent
## solutions for this shift position.

## Warning in params_process.PhyloEM(res, K = 6): There are several equivalent
## solutions for this shift position.

## Warning in params_process.PhyloEM(res, K = 6): There are several equivalent
## solutions for this shift position.

## Warning in params_process.PhyloEM(res, K = 6): There are several equivalent
## solutions for this shift position.

## Warning in params_process.PhyloEM(res, K = 6): There are several equivalent
## solutions for this shift position.

## Warning in params_process.PhyloEM(res, K = 6): There are several equivalent
## solutions for this shift position.

## Warning in params_process.PhyloEM(res, K = 6): There are several equivalent
## solutions for this shift position.

## Warning in params_process.PhyloEM(res, K = 6): There are several equivalent
## solutions for this shift position.

## Warning in params_process.PhyloEM(res, K = 6): There are several equivalent
## solutions for this shift position.
```

```
names(shiftTab)[1:4] <- c("primates","marsupials","perissodactyls","pinnipeds") 
rownames(shiftTab) <- c("Fat","Protein","Sugar")

shiftTabc <- shiftTab
for (i in 1:ncol(shiftTabc)){ shiftTabc[,i] <- encl(shiftTabc[,i], digits=5, brackets=c("","")) }
knitr::kable(shiftTabc)
```

|  | primates | marsupials | perissodactyls | pinnipeds | Mirounga | otariids |
| --- | --- | --- | --- | --- | --- | --- |
| Fat | 3.18224 | 6.83607 | 1.20587 | 59.78315 | 44.72456 | 43.04597 |
| Protein | 2.36287 | 7.95330 | 2.37018 | 7.97800 | 8.36046 | 10.83272 |
| Sugar | 7.18186 | 7.71032 | 6.06746 | 0.76505 | 0.00007 | 0.08824 |

```
cat(knitr::kable(shiftTabc, format="latex", booktabs=TRUE), file="output/phyloEMshiftTab.tex") ## for manuscript
```

Build something graphical to explain the best model.

```
gdat <- milk[,c("phyName","Species","itFat","itProtein","itSugar")]
names(gdat) <- gsub("it","",names(gdat))
```

We can get model imputed values (e.g. missing sugar observations), too.

```
impDat <- data.frame(phyName = consTree$tip.label,
         impFat = as.numeric(imputed_traits(res, trait=1, where="tips")),
         impProtein = as.numeric(imputed_traits(res, trait=2, where="tips")),
         impSugar = as.numeric(imputed_traits(res, trait=3, where="tips")))
```

```
## Warning in params_process.PhyloEM(x, method.selection, ...): There are
## several equivalent solutions for this shift position.

## Warning in params_process.PhyloEM(x, method.selection, ...): There are
## several equivalent solutions for this shift position.

## Warning in params_process.PhyloEM(x, method.selection, ...): There are
## several equivalent solutions for this shift position.
```

```
gdat <- merge(gdat, impDat, by="phyName")
gdat$iFat <- 1
gdat$iFat[is.na(gdat$Fat)==TRUE] <- 2
gdat$Fat[is.na(gdat$Fat)==TRUE] <- gdat$impFat[is.na(gdat$Fat)==TRUE]
gdat$iFat <- factor(gdat$iFat, levels=1:2, labels=c("obs","imp"))
gdat$iProtein <- 1
gdat$iProtein[is.na(gdat$Protein)==TRUE] <- 2
gdat$Protein[is.na(gdat$Protein)==TRUE] <- gdat$impProtein[is.na(gdat$Protein)==TRUE]
gdat$iProtein <- factor(gdat$iProtein, levels=1:2, labels=c("obs","imp"))
gdat$iSugar <- 1
gdat$iSugar[is.na(gdat$Sugar)==TRUE] <- 2
gdat$Sugar[is.na(gdat$Sugar)==TRUE] <- gdat$impSugar[is.na(gdat$Sugar)==TRUE]
gdat$iSugar <- factor(gdat$iSugar, levels=1:2, labels=c("obs","imp"))
table(gdat$iSugar)
```

```
## 
## obs imp 
## 109  15
```

Subtract the root value to “center” the data.

```
gdat$Fat <- gdat$Fat - params_process(res, K = 6)$root.state$exp.root[1]
```

```
## Warning in params_process.PhyloEM(res, K = 6): There are several equivalent
## solutions for this shift position.
```

```
gdat$Protein <- gdat$Protein - params_process(res, K = 6)$root.state$exp.root[2]
```

```
## Warning in params_process.PhyloEM(res, K = 6): There are several equivalent
## solutions for this shift position.
```

```
gdat$Sugar <- gdat$Sugar - params_process(res, K = 6)$root.state$exp.root[3]
```

```
## Warning in params_process.PhyloEM(res, K = 6): There are several equivalent
## solutions for this shift position.
```

Build up a plot. Use the milk data taxon labels.

```
SpconsTreeLab <- consTreeLab
SpconsTreeLab$tip.label <- as.character(gdat$Species)[match(consTreeLab$tip.label, gdat$phyName)]
SpconsTreeLab$tip.label[49] ## real taxon from milk data
```

```
## [1] "Cervus elaphus hispanicus"
```

```
consTreeLab$tip.label[49]   ## used in phylogeny
```

```
## [1] "Cervus_astylodon"
```

Complete phylogeny with node labels for reference. Useful for KSI results.

```
savepar <- par()
pdf(file="output/phylowithnodelabels-fan.pdf", height=12, width=12)
plot(SpconsTreeLab, show.node.label=TRUE, cex=.65, no.margin=TRUE, type="fan")
par(savepar)
```

```
## Warning in par(savepar): graphical parameter "cin" cannot be set
```

```
## Warning in par(savepar): graphical parameter "cra" cannot be set
```

```
## Warning in par(savepar): graphical parameter "csi" cannot be set
```

```
## Warning in par(savepar): graphical parameter "cxy" cannot be set
```

```
## Warning in par(savepar): graphical parameter "din" cannot be set
```

```
## Warning in par(savepar): graphical parameter "page" cannot be set
```

```
graphics.off()
```

Use `table.phylo4d` format for plotting `PhyloEM` results.

```
t4 <- as(SpconsTreeLab, "phylo4")
rownames(gdat) <- gdat$Species
phy4 <- phylo4d(t4, gdat[,c("Fat","Protein","Sugar")])
```

Downloaded phylopic icons with 50% transparency added.

```
macropus      <- readPNG('phylopics/macropus_c306572a-fae1-41e3-8208-c2bce972e0ef.512_o.png')
ateles        <- readPNG('phylopics/ateles_aceb287d-84cf-46f1-868c-4797c4ac54a8.512_o.png')
pagophilus    <- readPNG('phylopics/pagophilus_5e359baf-a5f7-4101-8f61-6d42beb52756.512_o.png')
arctocephalus <- readPNG('phylopics/arctocephalus_7ce9ff63-7eec-4fc3-8f37-5f66f9b924a9.512_o.png')
mirounga      <- readPNG('phylopics/tumblr_p4z5peLHSd1trl4uyo1_500_o.png') # none on phylopic, had to make this one
## https://the-faunal-frontier.tumblr.com/post/172382176896/mirounga-angustirostris-northern-elephant-seal was original source
zebra         <- readPNG('phylopics/zebra_a31e7527-3203-4233-b0da-c415cc7d1664.512_o.png')
```

Use a range of -6 to 3 for legend.

```
apply(gdat[c("Fat","Protein","Sugar")], 2, min)
```

```
##       Fat   Protein     Sugar 
## -4.221472 -2.004121 -5.228022
```

```
apply(gdat[c("Fat","Protein","Sugar")], 2, max)
```

```
##       Fat   Protein     Sugar 
## 2.4426521 0.8214935 1.4736810
```

Construct a display of the phylogeny with labeled tips, milk data
as shifts from PhyloEM() root and highlight the clades where shifts were inferred.

```
pdf(file="output/PhyloEMshifts.pdf", height=14.5, width=8)
par(mar=c(0,0,0,0)+.2)
gtablephylo4d(phy4, center=FALSE, scale=FALSE, box=FALSE, show.node.label=FALSE, grid=FALSE, ratio.tree=.78, legend=TRUE, cex.legend = 1, cex.label=.63, symbol="colors", pch=15, cex.symbol=1.6, coord.legend=list("x"=0, "y"=6.5), edge.color = "black", edge.width = 2.2, leg.vals = c(-6,-3,-2,-1,0,1,2,3), col = viridis(100)) #, col=brewer.pal(n=11, name="RdYlBu")  )
## icons on the clades
grid.raster(macropus,      .48,  .86,   width=.17)
grid.raster(ateles,        .52,  .65,   width=.12)
grid.raster(zebra,         .59,  .297,  width=.12)
grid.raster(pagophilus,    .66,  .183,  width=.14)
grid.raster(arctocephalus, .71,  .15,   width=.07)
grid.raster(mirounga,      .734, .225,  width=.03)
## icons in a legend
grid.raster(macropus,      .12,  .43,  width=.055)
grid.raster(ateles,        .12,  .40,  width=.04)
grid.raster(zebra,         .12,  .37,  width=.05)
grid.raster(pagophilus,    .12,  .34,  width=.05)
grid.raster(arctocephalus, .12,  .31,  width=.05)
grid.raster(mirounga,      .12,  .28,  width=.05)
## labels for the icon legend
grid.text(label="marsupials",     .16,  .43, just="left", gp=gpar(fontsize=10))
grid.text(label="primates",       .16,  .40, just="left", gp=gpar(fontsize=10))
grid.text(label="perissodactyls", .16,  .37, just="left", gp=gpar(fontsize=10))
grid.text(label="pinnipeds",      .16,  .34, just="left", gp=gpar(fontsize=10))
grid.text(label="otariids",       .16,  .31, just="left", gp=gpar(fontsize=10))
grid.text(label="Mirounga",       .16,  .28, just="left", gp=gpar(fontsize=10, fontface="italic"))
## label for icon legend
grid.text(label="inferred clade shifts", .12, .46, just="left", gp=gpar(fontsize=10, fontface="bold"))
## label for color legend
grid.text(label="inferred change from root (logit)", .05, .21, just="left", gp=gpar(fontsize=10, fontface="bold"))
graphics.off()
par(savepar)
```

```
## Warning in par(savepar): graphical parameter "cin" cannot be set
```

```
## Warning in par(savepar): graphical parameter "cra" cannot be set
```

```
## Warning in par(savepar): graphical parameter "csi" cannot be set
```

```
## Warning in par(savepar): graphical parameter "cxy" cannot be set
```

```
## Warning in par(savepar): graphical parameter "din" cannot be set
```

```
## Warning in par(savepar): graphical parameter "page" cannot be set
```

```
graphics.off()
```

# Ecological Predictors and Multivariate Phylogenetic Signal

*WARNING* Running this model is slow. The results from a new run
will not exactly match those reported in the manuscript because
each `MCMCglmm()` run is different, even with `set.seed()`.

```
set.seed(1234)
```

Set priors for MCMCglmm model.
Use inverse Wishart for residual ® and parameter expanded for phylogeny (G).

```
prs3 <- list(R=list(V=diag(3), nu=.002),
                  G=list(
                    G1=list(V=diag(3), nu=3, 
                            alpha.mu=rep(0,3), alpha.V=diag(3)*1000)))
```

Use logit-transformed milk compositions.
Use all the ecological predictors (log-transformed and centered if continuous) and the phylogeny.

```
(10050000 - 50000) / 2000 ## control iteration, should give effective sample size close to 5000
```

```
## [1] 5000
```

```
milk$animal <- milk$phyName ## mandatory name
system.time( mcCovPhyAllItDat <- MCMCglmm(cbind(itFat,itProtein,itSugar) ~  trait + Arid*trait + ordAq*trait + Diet*trait + Female.mass*trait + Repro.output*trait + RelLact.length*trait + Developmental.stage.at.birth*trait - 1, random = ~us(trait):animal, rcov = ~us(trait):units, data=milk, prior=prs3, family=rep("gaussian",3), pl=TRUE, pedigree = consTree, nitt=10050000, burnin=50000, thin=2000, verbose=FALSE) )
```

```
##     user   system  elapsed 
## 8981.560    0.364 8982.308
```

Check for good posteriors.

```
summary(mcCovPhyAllItDat)
```

```
## 
##  Iterations = 50001:10048001
##  Thinning interval  = 2000
##  Sample size  = 5000 
## 
##  DIC: -202.2103 
## 
##  G-structure:  ~us(trait):animal
## 
##                                      post.mean l-95% CI u-95% CI eff.samp
## traititFat:traititFat.animal            2.6427   1.5880  3.87024     5000
## traititProtein:traititFat.animal        1.0677   0.5725  1.56505     4601
## traititSugar:traititFat.animal         -1.3394  -2.2185 -0.50892     5000
## traititFat:traititProtein.animal        1.0677   0.5725  1.56505     4601
## traititProtein:traititProtein.animal    1.0591   0.6996  1.41851     5000
## traititSugar:traititProtein.animal     -0.5042  -0.9147 -0.08538     5000
## traititFat:traititSugar.animal         -1.3394  -2.2185 -0.50892     5000
## traititProtein:traititSugar.animal     -0.5042  -0.9147 -0.08538     5000
## traititSugar:traititSugar.animal        1.4143   0.4431  2.64005     5000
## 
##  R-structure:  ~us(trait):units
## 
##                                     post.mean   l-95% CI u-95% CI eff.samp
## traititFat:traititFat.units         0.0799866  0.0227576  0.13822     4751
## traititProtein:traititFat.units     0.0008558 -0.0170127  0.01933     5000
## traititSugar:traititFat.units       0.0495441 -0.0185807  0.11971     5000
## traititFat:traititProtein.units     0.0008558 -0.0170127  0.01933     5000
## traititProtein:traititProtein.units 0.0079161  0.0004024  0.01816     5000
## traititSugar:traititProtein.units   0.0066592 -0.0246803  0.03631     5000
## traititFat:traititSugar.units       0.0495441 -0.0185807  0.11971     5000
## traititProtein:traititSugar.units   0.0066592 -0.0246803  0.03631     5000
## traititSugar:traititSugar.units     0.2199239  0.1012281  0.35946     5000
## 
##  Location effects: cbind(itFat, itProtein, itSugar) ~ trait + Arid * trait + ordAq * trait + Diet * trait + Female.mass * trait + Repro.output * trait + RelLact.length * trait + Developmental.stage.at.birth * trait - 1 
## 
##                                             post.mean l-95% CI u-95% CI
## traititFat                                   -2.16082 -3.94550 -0.31725
## traititProtein                               -2.34722 -3.44751 -1.25217
## traititSugar                                 -3.20353 -4.59863 -1.80504
## Arid                                         -0.19023 -0.50717  0.14143
## ordAq                                         0.22011 -0.14571  0.62133
## Dietomnivore                                  0.19374 -0.19747  0.57012
## Dietcarnivore                                 0.79920  0.22566  1.39644
## Female.mass                                  -0.04934 -0.26247  0.17543
## Repro.output                                  0.14989 -0.17844  0.51864
## RelLact.length                               -0.90051 -1.62053 -0.22031
## Developmental.stage.at.birth                  0.06622 -0.12740  0.26391
## traititProtein:Arid                           0.02450 -0.25665  0.30345
## traititSugar:Arid                             0.17090 -0.33201  0.69869
## traititProtein:ordAq                         -0.17620 -0.51374  0.13145
## traititSugar:ordAq                           -0.59655 -1.25436  0.01218
## traititProtein:Dietomnivore                  -0.22246 -0.53712  0.11084
## traititSugar:Dietomnivore                    -0.10476 -0.71194  0.51992
## traititProtein:Dietcarnivore                 -0.63688 -1.14262 -0.13048
## traititSugar:Dietcarnivore                   -1.17500 -2.21142 -0.21620
## traititProtein:Female.mass                    0.01535 -0.16722  0.20749
## traititSugar:Female.mass                     -0.05395 -0.42433  0.27918
## traititProtein:Repro.output                   0.01739 -0.30705  0.34560
## traititSugar:Repro.output                    -0.39592 -0.95718  0.17884
## traititProtein:RelLact.length                 1.00532  0.31808  1.60576
## traititSugar:RelLact.length                   1.29803  0.05013  2.42583
## traititProtein:Developmental.stage.at.birth  -0.09712 -0.26663  0.07477
## traititSugar:Developmental.stage.at.birth    -0.10491 -0.42438  0.20969
##                                             eff.samp  pMCMC    
## traititFat                                      5239 0.0244 *  
## traititProtein                                  5385 <2e-04 ***
## traititSugar                                    5000 <2e-04 ***
## Arid                                            5000 0.2552    
## ordAq                                           5000 0.2596    
## Dietomnivore                                    5000 0.3244    
## Dietcarnivore                                   5226 0.0068 ** 
## Female.mass                                     5000 0.6516    
## Repro.output                                    5000 0.3720    
## RelLact.length                                  5000 0.0132 *  
## Developmental.stage.at.birth                    5000 0.5000    
## traititProtein:Arid                             5000 0.8652    
## traititSugar:Arid                               5000 0.5252    
## traititProtein:ordAq                            5000 0.2796    
## traititSugar:ordAq                              5000 0.0656 .  
## traititProtein:Dietomnivore                     5000 0.1744    
## traititSugar:Dietomnivore                       5000 0.7400    
## traititProtein:Dietcarnivore                    5000 0.0120 *  
## traititSugar:Dietcarnivore                      5000 0.0228 *  
## traititProtein:Female.mass                      5000 0.8660    
## traititSugar:Female.mass                        5000 0.7616    
## traititProtein:Repro.output                     5000 0.9144    
## traititSugar:Repro.output                       5000 0.1692    
## traititProtein:RelLact.length                   5000 0.0020 ** 
## traititSugar:RelLact.length                     5000 0.0344 *  
## traititProtein:Developmental.stage.at.birth     4941 0.2548    
## traititSugar:Developmental.stage.at.birth       4433 0.4992    
## ---
## Signif. codes:  0 '***' 0.001 '**' 0.01 '*' 0.05 '.' 0.1 ' ' 1
```

```
effectiveSize(mcCovPhyAllItDat$VCV) ## looks good
```

```
##         traititFat:traititFat.animal     traititProtein:traititFat.animal 
##                             5000.000                             4600.647 
##       traititSugar:traititFat.animal     traititFat:traititProtein.animal 
##                             5000.000                             4600.647 
## traititProtein:traititProtein.animal   traititSugar:traititProtein.animal 
##                             5000.000                             5000.000 
##       traititFat:traititSugar.animal   traititProtein:traititSugar.animal 
##                             5000.000                             5000.000 
##     traititSugar:traititSugar.animal          traititFat:traititFat.units 
##                             5000.000                             4751.481 
##      traititProtein:traititFat.units        traititSugar:traititFat.units 
##                             5000.000                             5000.000 
##      traititFat:traititProtein.units  traititProtein:traititProtein.units 
##                             5000.000                             5000.000 
##    traititSugar:traititProtein.units        traititFat:traititSugar.units 
##                             5000.000                             5000.000 
##    traititProtein:traititSugar.units      traititSugar:traititSugar.units 
##                             5000.000                             5000.000
```

```
autocorr.diag(mcCovPhyAllItDat$VCV) ## ditto
```

```
##           traititFat:traititFat.animal traititProtein:traititFat.animal
## Lag 0                     1.0000000000                     1.0000000000
## Lag 2000                  0.0136978321                    -0.0007024977
## Lag 10000                 0.0003098685                     0.0313509392
## Lag 20000                 0.0117857836                     0.0348881108
## Lag 1e+05                 0.0242857695                     0.0320932503
##           traititSugar:traititFat.animal traititFat:traititProtein.animal
## Lag 0                       1.0000000000                     1.0000000000
## Lag 2000                    0.0067164544                    -0.0007024977
## Lag 10000                   0.0009806374                     0.0313509392
## Lag 20000                   0.0201764286                     0.0348881108
## Lag 1e+05                  -0.0158987565                     0.0320932503
##           traititProtein:traititProtein.animal
## Lag 0                              1.000000000
## Lag 2000                          -0.012294840
## Lag 10000                         -0.013640077
## Lag 20000                          0.024891228
## Lag 1e+05                          0.008741839
##           traititSugar:traititProtein.animal
## Lag 0                            1.000000000
## Lag 2000                        -0.001086500
## Lag 10000                       -0.001222089
## Lag 20000                        0.037629020
## Lag 1e+05                        0.017236616
##           traititFat:traititSugar.animal
## Lag 0                       1.0000000000
## Lag 2000                    0.0067164544
## Lag 10000                   0.0009806374
## Lag 20000                   0.0201764286
## Lag 1e+05                  -0.0158987565
##           traititProtein:traititSugar.animal
## Lag 0                            1.000000000
## Lag 2000                        -0.001086500
## Lag 10000                       -0.001222089
## Lag 20000                        0.037629020
## Lag 1e+05                        0.017236616
##           traititSugar:traititSugar.animal traititFat:traititFat.units
## Lag 0                          1.000000000                 1.000000000
## Lag 2000                       0.001335038                 0.025385290
## Lag 10000                     -0.005821107                -0.003455585
## Lag 20000                      0.009503112                 0.010915807
## Lag 1e+05                     -0.028738797                 0.001332722
##           traititProtein:traititFat.units traititSugar:traititFat.units
## Lag 0                         1.000000000                   1.000000000
## Lag 2000                      0.005749083                   0.001153775
## Lag 10000                     0.002505283                  -0.010182587
## Lag 20000                     0.001437951                  -0.004133260
## Lag 1e+05                     0.017029300                  -0.021710087
##           traititFat:traititProtein.units
## Lag 0                         1.000000000
## Lag 2000                      0.005749083
## Lag 10000                     0.002505283
## Lag 20000                     0.001437951
## Lag 1e+05                     0.017029300
##           traititProtein:traititProtein.units
## Lag 0                             1.000000000
## Lag 2000                         -0.018783381
## Lag 10000                        -0.021933868
## Lag 20000                         0.017214387
## Lag 1e+05                         0.007385951
##           traititSugar:traititProtein.units traititFat:traititSugar.units
## Lag 0                           1.000000000                   1.000000000
## Lag 2000                        0.003468206                   0.001153775
## Lag 10000                       0.015818865                  -0.010182587
## Lag 20000                       0.007468420                  -0.004133260
## Lag 1e+05                       0.024954468                  -0.021710087
##           traititProtein:traititSugar.units
## Lag 0                           1.000000000
## Lag 2000                        0.003468206
## Lag 10000                       0.015818865
## Lag 20000                       0.007468420
## Lag 1e+05                       0.024954468
##           traititSugar:traititSugar.units
## Lag 0                         1.000000000
## Lag 2000                      0.014839839
## Lag 10000                     0.013878536
## Lag 20000                    -0.010672115
## Lag 1e+05                    -0.004477567
```

Can also check trace and density plots of variance component posteriors.

```
## plot(mcCovPhyAllItDat$VCV)
```

Get posterior distributions for the phylogenetic and residual
correlations and the phylogenetic “heritabilities.”

```
rA_fp <- mcCovPhyAllItDat$VCV[,"traititFat:traititProtein.animal"] / sqrt(mcCovPhyAllItDat$VCV[,"traititFat:traititFat.animal"] *  mcCovPhyAllItDat$VCV[,"traititProtein:traititProtein.animal"] )
rA_fs <- mcCovPhyAllItDat$VCV[,"traititFat:traititSugar.animal"] / sqrt(mcCovPhyAllItDat$VCV[,"traititFat:traititFat.animal"] *  mcCovPhyAllItDat$VCV[,"traititSugar:traititSugar.animal"] )

rA_ps <- mcCovPhyAllItDat$VCV[,"traititSugar:traititProtein.animal"] / sqrt(mcCovPhyAllItDat$VCV[,"traititSugar:traititSugar.animal"] *  mcCovPhyAllItDat$VCV[,"traititProtein:traititProtein.animal"] )


rR_fp <- mcCovPhyAllItDat$VCV[,"traititFat:traititProtein.units"] / sqrt(mcCovPhyAllItDat$VCV[,"traititFat:traititFat.units"] *  mcCovPhyAllItDat$VCV[,"traititProtein:traititProtein.units"] )
rR_fs <- mcCovPhyAllItDat$VCV[,"traititFat:traititSugar.units"] / sqrt(mcCovPhyAllItDat$VCV[,"traititFat:traititFat.units"] *  mcCovPhyAllItDat$VCV[,"traititSugar:traititSugar.units"] )

rR_ps <- mcCovPhyAllItDat$VCV[,"traititSugar:traititProtein.units"] / sqrt(mcCovPhyAllItDat$VCV[,"traititSugar:traititSugar.units"] *  mcCovPhyAllItDat$VCV[,"traititProtein:traititProtein.units"] )


h2_f <- mcCovPhyAllItDat$VCV[,"traititFat:traititFat.animal"] / (mcCovPhyAllItDat$VCV[,"traititFat:traititFat.units"] +  mcCovPhyAllItDat$VCV[,"traititFat:traititFat.animal"] )
h2_p <- mcCovPhyAllItDat$VCV[,"traititProtein:traititProtein.animal"] / (mcCovPhyAllItDat$VCV[,"traititProtein:traititProtein.animal"] +  mcCovPhyAllItDat$VCV[,"traititProtein:traititProtein.units"] )
h2_s <- mcCovPhyAllItDat$VCV[,"traititSugar:traititSugar.animal"] / (mcCovPhyAllItDat$VCV[,"traititSugar:traititSugar.units"] +  mcCovPhyAllItDat$VCV[,"traititSugar:traititSugar.animal"] )
```

Check for good posteriors on these ratios, too.

```
effectiveSize(cbind(rA_fp, rA_fs, rA_ps))
```

```
##    rA_fp    rA_fs    rA_ps 
## 3944.145 5000.000 5000.000
```

```
effectiveSize(cbind(rR_fp, rR_fs, rR_ps))
```

```
##    rR_fp    rR_fs    rR_ps 
## 5000.000 5000.000 4526.051
```

```
effectiveSize(cbind(h2_f, h2_p, h2_s))
```

```
##     h2_f     h2_p     h2_s 
## 4758.648 5000.000 5000.000
```

```
plot(rA_fs) ## needed long run to get rid of trends
```

```
plot(h2_s)  ## ditto
```

```
autocorr.diag(rA_fs) ## 2k probably good
```

```
##                   [,1]
## Lag 0      1.000000000
## Lag 2000   0.003403265
## Lag 10000 -0.010636670
## Lag 20000  0.011089693
## Lag 1e+05  0.002537314
```

Assemble 3 x 3 matrix of:
rA - genetic/phylogenetic correlations above diagonal
rR - residual correlations below diagonal
h2 - phylogenetic signal on the diagonal
Use 3 separate matrices for posterior mode, low CI, and high CI.

```
corMat <- matrix(NA, 3,3)
corMatlo <- matrix(NA, 3,3)
corMathi <- matrix(NA, 3,3)
rownames(corMat) <- c("Fat","Protein","Sugar")
colnames(corMat) <- rownames(corMat)
rownames(corMatlo) <- rownames(corMat)
colnames(corMatlo) <- rownames(corMat)
rownames(corMathi) <- rownames(corMat)
colnames(corMathi) <- rownames(corMat)
```

Hard-coded putting rAs in upper triangle, rRs in lower and h2s on diagonal.

```
pmAs <- posterior.mode(as.mcmc(cbind(rA_fp, rA_fs,  rA_ps)), bw="SJ", adjust=1)
pmRs <- posterior.mode(as.mcmc(cbind(rR_fp, rR_fs,  rR_ps)), bw="SJ", adjust=1)
loAs <- HPDinterval(as.mcmc(cbind(rA_fp, rA_fs,  rA_ps)) )[,1]
loRs <- HPDinterval(as.mcmc(cbind(rR_fp, rR_fs,  rR_ps)) )[,1]
hiAs <- HPDinterval(as.mcmc(cbind(rA_fp, rA_fs,  rA_ps)) )[,2]
hiRs <- HPDinterval(as.mcmc(cbind(rR_fp, rR_fs,  rR_ps)) )[,2]
corMat[1,2:3] <- pmAs[1:2]
corMat[2,3] <- pmAs[3]
corMat[2:3,1] <- pmRs[1:2]
corMat[3,2] <- pmRs[3]

corMatlo[1,2:3] <- loAs[1:2]
corMatlo[2,3] <- loAs[3]
corMatlo[2:3,1] <- loRs[1:2]
corMatlo[3,2] <- loRs[3]

corMathi[1,2:3] <- hiAs[1:2]
corMathi[2,3] <- hiAs[3]
corMathi[2:3,1] <- hiRs[1:2]
corMathi[3,2] <- hiRs[3]

diag(corMat) <- posterior.mode(as.mcmc(cbind(h2_f, h2_p, h2_s)), bw="SJ", adjust=1)
diag(corMatlo) <- HPDinterval(as.mcmc(cbind(h2_f, h2_p, h2_s)))[,1]
diag(corMathi) <- HPDinterval(as.mcmc(cbind(h2_f, h2_p, h2_s)))[,2]

## corMat
## corMatlo
## corMathi
```

Assemble table with rounded values for manuscript.

```
rciTab <- matrix("", 3, 3)
rownames(rciTab) <- rownames(corMat)
colnames(rciTab) <- rownames(corMat)
for (i in 1:nrow(rciTab)){
    rciTab[,i] <- paste0(encl(corMat[,i],brackets=c(""," ")),
                         encl(corMatlo[,i],brackets=c("(",",")),
                         encl(corMathi[,i],brackets=c("",")")) )
    }

knitr::kable(rciTab)
```

|  | Fat | Protein | Sugar |
| --- | --- | --- | --- |
| Fat | 0.976 (0.934,0.994) | 0.675 (0.447,0.815) | -0.750 (-0.92,-0.464) |
| Protein | 0.156 (-0.775,0.688) | 0.997 (0.980,1.000) | -0.473 (-0.70,-0.119) |
| Sugar | 0.380 (-0.069,0.994) | 0.251 (-0.536,0.898) | 0.872 (0.68,0.979) |

```
cat(knitr::kable(rciTab, format="latex", booktabs=TRUE), file="output/mcModelcorMat.tex") ## basis for manuscript table
```

Find the regression coefficients for each milk variable.

```
rownames(summary(mcCovPhyAllItDat)$solutions)[c(1,4:9)]
```

```
## [1] "traititFat"    "Arid"          "ordAq"         "Dietomnivore" 
## [5] "Dietcarnivore" "Female.mass"   "Repro.output"
```

```
prots <- grep("Protein", rownames(summary(mcCovPhyAllItDat)$solutions))
sugs <- grep("Sugar", rownames(summary(mcCovPhyAllItDat)$solutions))
fats <- seq(1,ncol(mcCovPhyAllItDat$Sol))[-c(prots,sugs)]
## fats
## prots
## sugs
```

To get interpretable coefficients you need to add the protein and sugar ones onto fat,
but must keep the trait's intercept.

```
f0 <- mcCovPhyAllItDat$Sol[,fats]
f0[,1] <- 0
coefpms <- cbind(colMeans(mcCovPhyAllItDat$Sol[,fats]),
                 colMeans(mcCovPhyAllItDat$Sol[,prots] + f0),
                 colMeans(mcCovPhyAllItDat$Sol[,sugs] + f0) )
coeflos <- cbind(HPDinterval(mcCovPhyAllItDat$Sol[,fats])[,1],
                 HPDinterval(mcCovPhyAllItDat$Sol[,prots] + f0)[,1],
                 HPDinterval(mcCovPhyAllItDat$Sol[,sugs] + f0)[,1] )
coefhis <- cbind(HPDinterval(mcCovPhyAllItDat$Sol[,fats])[,2],
                 HPDinterval(mcCovPhyAllItDat$Sol[,prots] + f0)[,2],
                 HPDinterval(mcCovPhyAllItDat$Sol[,sugs] + f0)[,2] )
coefTab <- cbind(coefpms[,1], coeflos[,1], coefhis[,1],
                 coefpms[,2], coeflos[,2], coefhis[,2],
                 coefpms[,3], coeflos[,3], coefhis[,3] )
##coefTab
rownames(coefTab)[c(1,nrow(coefTab))] <- c("Intercept","Precociality")
colnames(coefTab) <- c("Fat","lfat","hfat", "Protein", 'lpro', 'hpro', "Sugar", 'lsug', 'hsug')
coefTab <- data.frame(coefTab)
##coefTab


coefPost <- cbind(mcCovPhyAllItDat$Sol[,fats],
                  mcCovPhyAllItDat$Sol[,prots] + f0,
                  mcCovPhyAllItDat$Sol[,sugs] + f0 )
##head(coefPost)
pMCs <- apply(coefPost, 2, function(z){
    g0x2 <- 2 * length(z[z>0])/length(z)
    l0x2 <- 2 * length(z[z<0])/length(z)
    min(c(g0x2,l0x2))
})
pMCmat <- matrix(pMCs, byrow=FALSE, ncol=3, nrow=nrow(coefTab))

coefTabp <- coefTab
for (i in seq(1,ncol(coefTab), by=3) ) {
    if (i ==1) {k=1}
    if (i ==4) {k=2}
    if (i ==7) {k=3}    
    coefTabp[,i] <- paste0(encl(coefTab[,i],brackets=c(""," ")),
                          encl(coefTab[,i+1],brackets=c("(",",")),
                          encl(coefTab[,i+2],brackets=c("",")")),
                          encl(pMCmat[,k],brackets=c(" ","")))
}
##coefTabp
coefTabp <- coefTabp[,seq(1,ncol(coefTab),by=3)]
##coefTabp

knitr::kable(coefTabp)
```

|  | Fat | Protein | Sugar |
| --- | --- | --- | --- |
| Intercept | -2.161 (-3.945,-0.317) 0.024 | -2.347 (-3.448,-1.252) 0.000 | -3.204 (-4.599,-1.805) 0.000 |
| Arid | -0.190 (-0.507,0.141) 0.255 | -0.166 (-0.355,0.022) 0.083 | -0.019 (-0.345,0.310) 0.925 |
| ordAq | 0.220 (-0.146,0.621) 0.260 | 0.044 (-0.174,0.268) 0.680 | -0.376 (-0.734,-0.014) 0.049 |
| Dietomnivore | 0.194 (-0.197,0.570) 0.324 | -0.029 (-0.248,0.191) 0.797 | 0.089 (-0.277,0.484) 0.648 |
| Dietcarnivore | 0.799 (0.226,1.396) 0.007 | 0.162 (-0.173,0.490) 0.326 | -0.376 (-0.978,0.289) 0.245 |
| Female.mass | -0.049 (-0.262,0.175) 0.652 | -0.034 (-0.152,0.090) 0.562 | -0.103 (-0.298,0.103) 0.313 |
| Repro.output | 0.150 (-0.178,0.519) 0.372 | 0.167 (0.001,0.344) 0.053 | -0.246 (-0.624,0.136) 0.199 |
| RelLact.length | -0.901 (-1.621,-0.220) 0.013 | 0.105 (-0.269,0.479) 0.595 | 0.398 (-0.443,1.227) 0.338 |
| Precociality | 0.066 (-0.127,0.264) 0.500 | -0.031 (-0.139,0.088) 0.598 | -0.039 (-0.234,0.164) 0.703 |

```
cat(knitr::kable(coefTabp, format="latex", booktabs=TRUE), file="output/mcModelcoefs.tex") ## massage for manuscript
```

# Session Information

```
sessionInfo()
```

```
## R version 3.5.2 (2018-12-20)
## Platform: x86_64-pc-linux-gnu (64-bit)
## Running under: Debian GNU/Linux 10 (buster)
## 
## Matrix products: default
## BLAS: /usr/lib/x86_64-linux-gnu/atlas/libblas.so.3.10.3
## LAPACK: /usr/lib/x86_64-linux-gnu/atlas/liblapack.so.3.10.3
## 
## locale:
##  [1] LC_CTYPE=en_US.UTF-8       LC_NUMERIC=C              
##  [3] LC_TIME=en_US.UTF-8        LC_COLLATE=en_US.UTF-8    
##  [5] LC_MONETARY=en_US.UTF-8    LC_MESSAGES=en_US.UTF-8   
##  [7] LC_PAPER=en_US.UTF-8       LC_NAME=C                 
##  [9] LC_ADDRESS=C               LC_TELEPHONE=C            
## [11] LC_MEASUREMENT=en_US.UTF-8 LC_IDENTIFICATION=C       
## 
## attached base packages:
## [1] grid      stats     graphics  grDevices utils     datasets  methods  
## [8] base     
## 
## other attached packages:
##  [1] Peacock.test_1.0     ggrepel_0.8.1        png_0.1-7           
##  [4] viridis_0.5.1        viridisLite_0.3.0    adephylo_1.1-11     
##  [7] ade4_1.7-13          phylobase_0.8.6      reshape2_1.4.3      
## [10] gridExtra_2.3        ggplot2_3.1.0        phytools_0.6-60     
## [13] maps_3.3.0           PhylogeneticEM_1.2.1 MCMCglmm_2.28       
## [16] ape_5.2              coda_0.19-2          Matrix_1.2-15       
## [19] ksi_0.1-3           
## 
## loaded via a namespace (and not attached):
##  [1] cubature_2.0.3          colorspace_1.4-0       
##  [3] seqinr_3.4-5            deldir_0.1-16          
##  [5] corpcor_1.6.9           pls_2.7-0              
##  [7] mvtnorm_1.0-10          xml2_1.2.0             
##  [9] codetools_0.2-16        splines_3.5.2          
## [11] mnormt_1.5-5            doParallel_1.0.14      
## [13] knitr_1.21              cluster_2.0.7-1        
## [15] shiny_1.2.0             compiler_3.5.2         
## [17] httr_1.4.0              assertthat_0.2.0       
## [19] lazyeval_0.2.1          later_0.7.5            
## [21] lars_1.2                htmltools_0.3.6        
## [23] prettyunits_1.0.2       tools_3.5.2            
## [25] bindrcpp_0.2.2          igraph_1.2.4           
## [27] gtable_0.2.0            glue_1.3.0             
## [29] clusterGeneration_1.3.4 dplyr_0.7.8            
## [31] gmodels_2.18.1          fastmatch_1.1-0        
## [33] Rcpp_1.0.0              spdep_0.8-1            
## [35] gdata_2.18.0            nlme_3.1-137           
## [37] iterators_1.0.10        tensorA_0.36.1         
## [39] xfun_0.4                stringr_1.4.0          
## [41] mime_0.6                phangorn_2.5.3         
## [43] gtools_3.8.1            XML_3.98-1.17          
## [45] LearnBayes_2.15.1       MASS_7.3-51.1          
## [47] scales_1.0.0            LINselect_1.1          
## [49] subplex_1.5-4           hms_0.4.2              
## [51] promises_1.0.1          parallel_3.5.2         
## [53] expm_0.999-4            animation_2.6          
## [55] elasticnet_1.1.1        diversitree_0.9-11     
## [57] capushe_1.1.1           stringi_1.2.4          
## [59] highr_0.7               foreach_1.4.4          
## [61] plotrix_3.7-5           randomForest_4.6-14    
## [63] permute_0.9-5           boot_1.3-20            
## [65] spData_0.3.0            rlang_0.3.1            
## [67] pkgconfig_2.0.2         rncl_0.8.3             
## [69] evaluate_0.13           lattice_0.20-38        
## [71] purrr_0.3.0             bindr_0.1.1            
## [73] labeling_0.3            tidyselect_0.2.5       
## [75] deSolve_1.21            plyr_1.8.4             
## [77] magrittr_1.5            R6_2.4.0               
## [79] combinat_0.0-8          pillar_1.3.1           
## [81] withr_2.1.2             mgcv_1.8-27            
## [83] scatterplot3d_0.3-41    sp_1.3-1               
## [85] tibble_2.0.1            crayon_1.3.4           
## [87] uuid_0.1-2              progress_1.2.0         
## [89] RNeXML_2.3.0            adegenet_2.1.1         
## [91] vegan_2.5-4             digest_0.6.18          
## [93] xtable_1.8-3            tidyr_0.8.2            
## [95] httpuv_1.4.5.1          numDeriv_2016.8-1      
## [97] munsell_0.5.0           quadprog_1.5-5
```

```
date()
```

```
## [1] "Sun Sep 15 17:46:19 2019"
```
