# Supplementary figures and images for: Adaptation, phylogeny, and covariance in milk macronutrient composition
Source: PeerJ. 2019 Nov 13;7:e8085. doi: 10.7717/peerj.8085 (PMC6858816; doi:10.7717/peerj.8085)

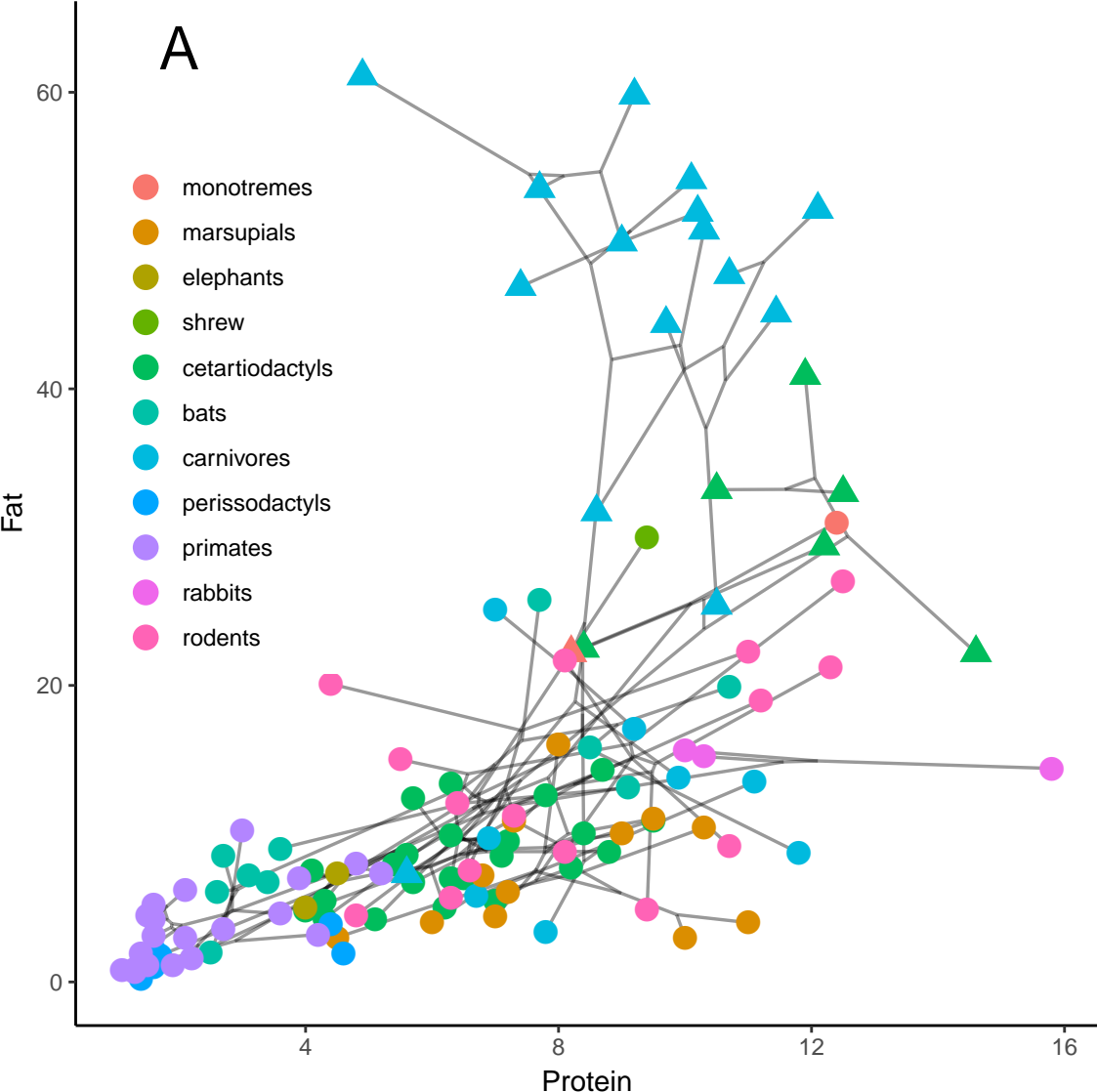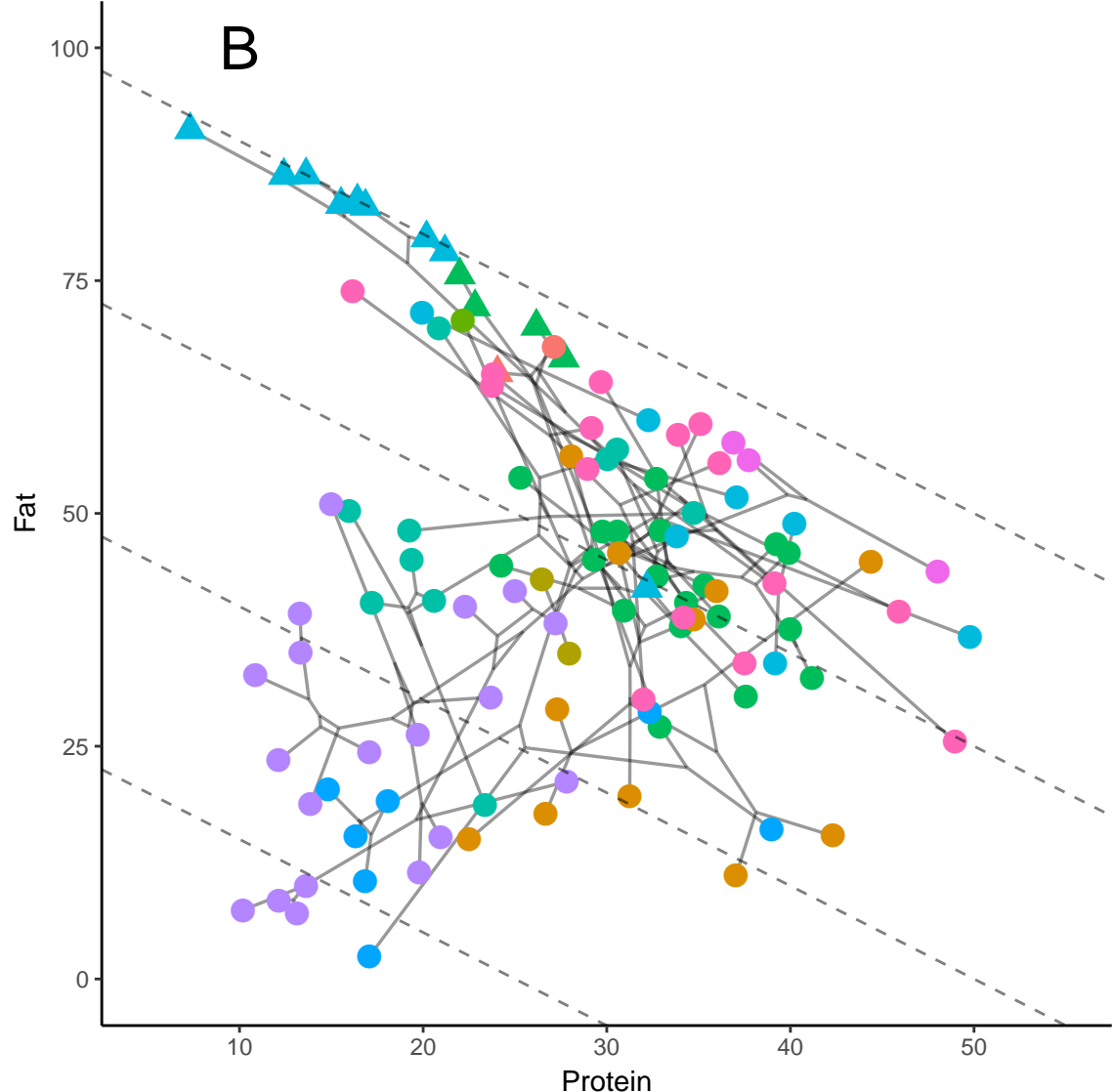

Supplement: Supplemental Information 1 [file peerj-07-8085-s001.zip › output/Figure1.pdf]

# clade shift selection criteria

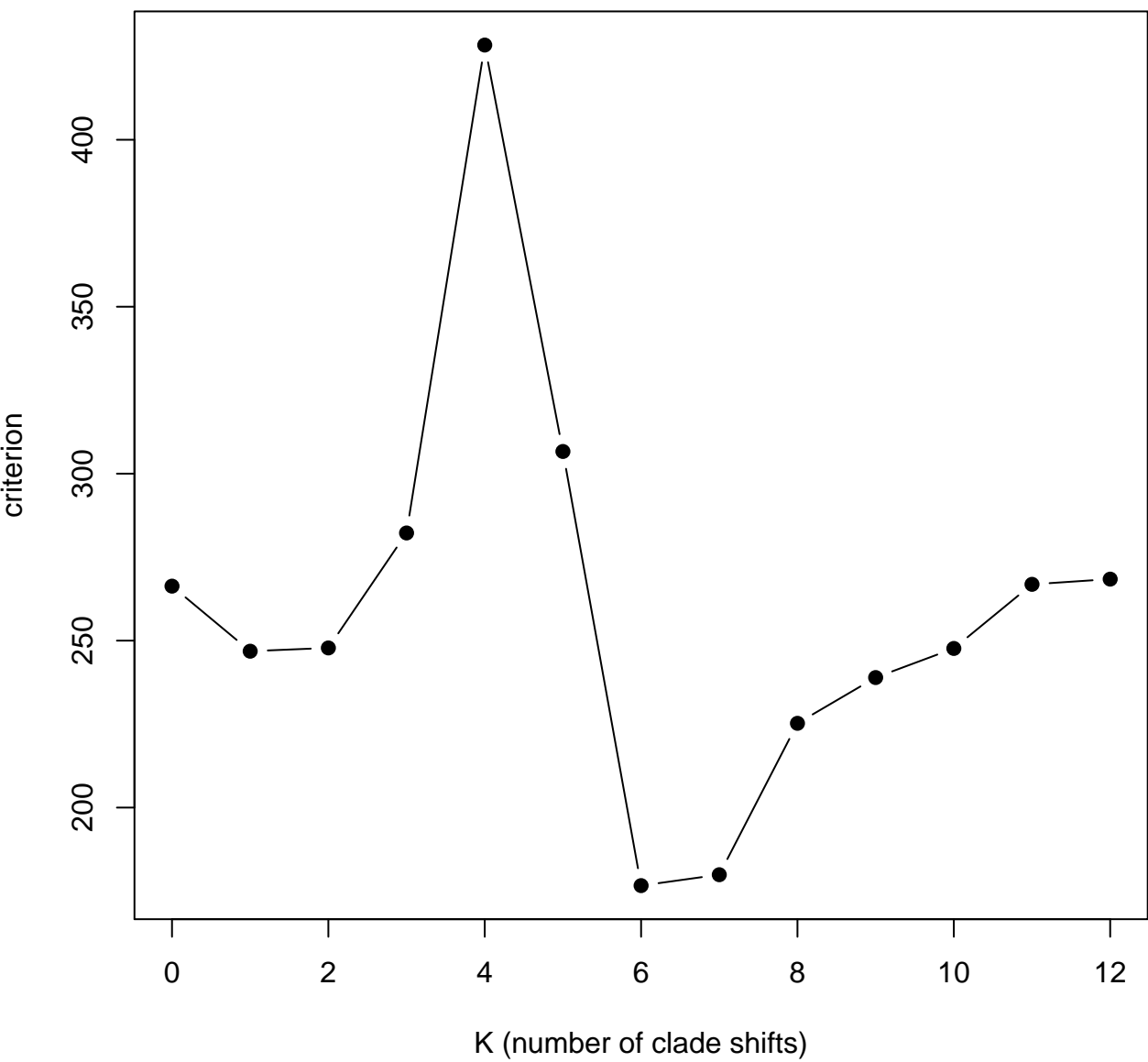

Supplement: Supplemental Information 1 [file peerj-07-8085-s001.zip › output/Figure2.pdf]

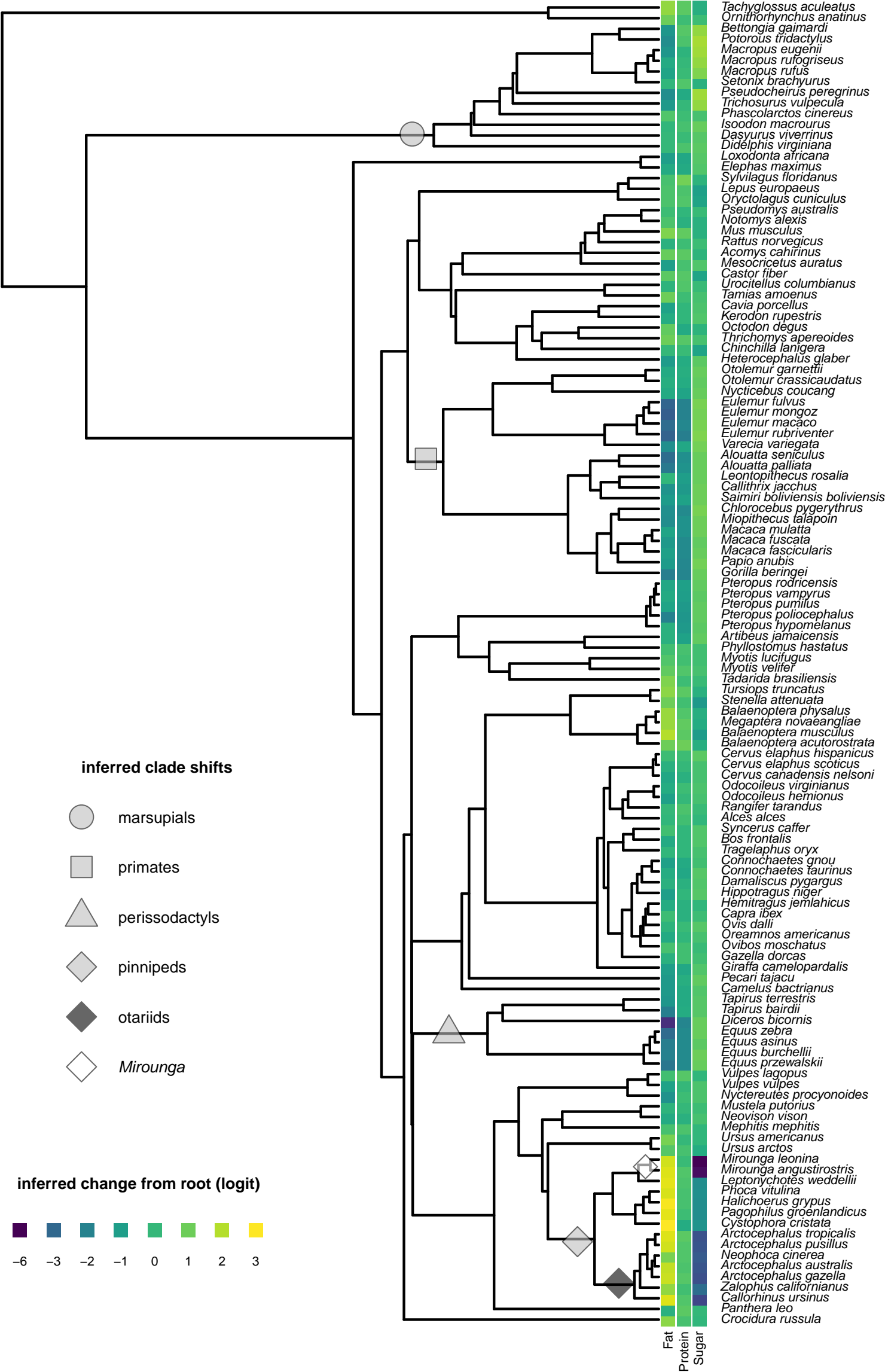

Supplement: Supplemental Information 1 [file peerj-07-8085-s001.zip › output/Figure3.pdf]

# clade shift selection criteria

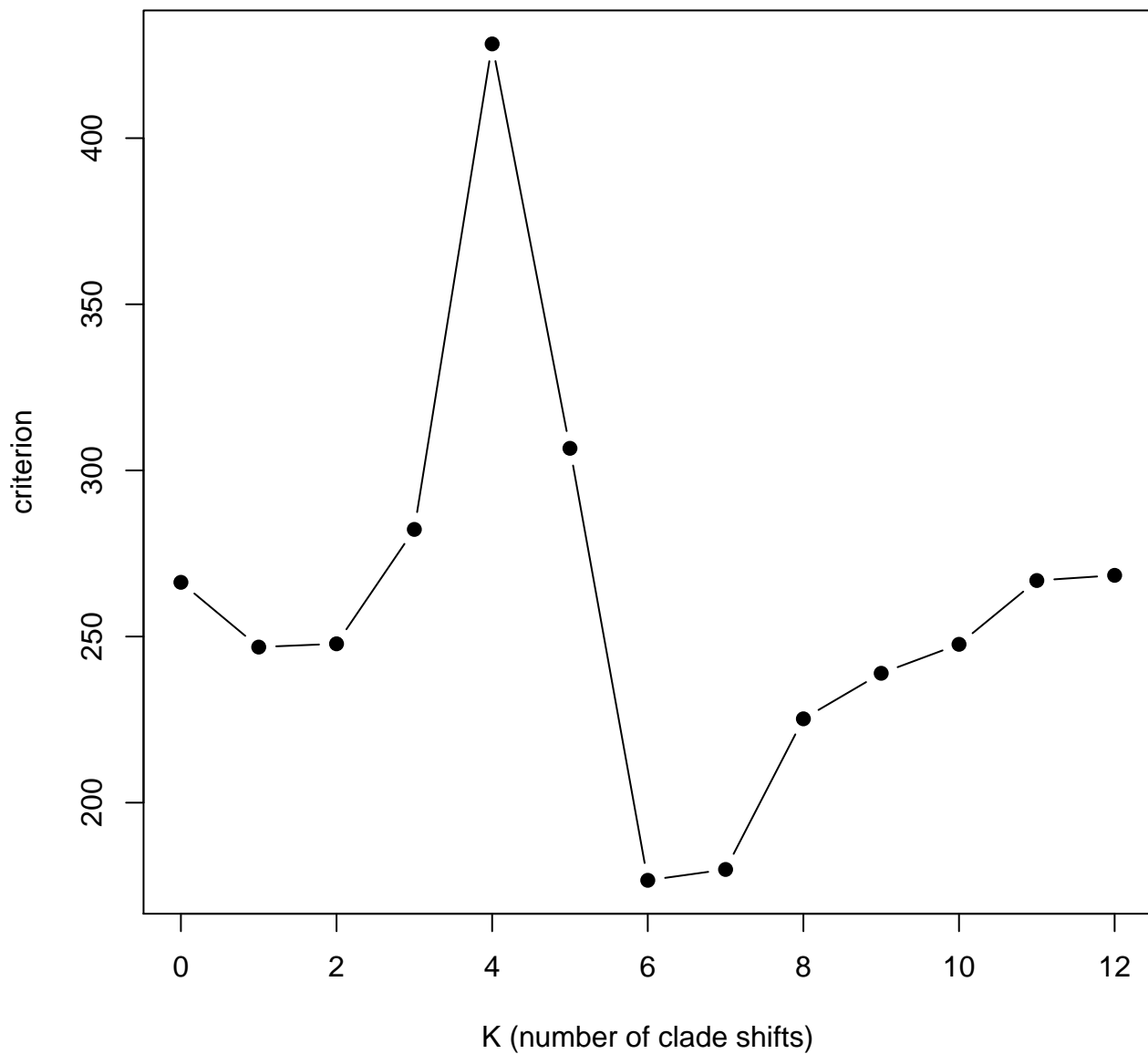

Supplement: Supplemental Information 1 [file peerj-07-8085-s001.zip › output/PhyloEMselectK.pdf]

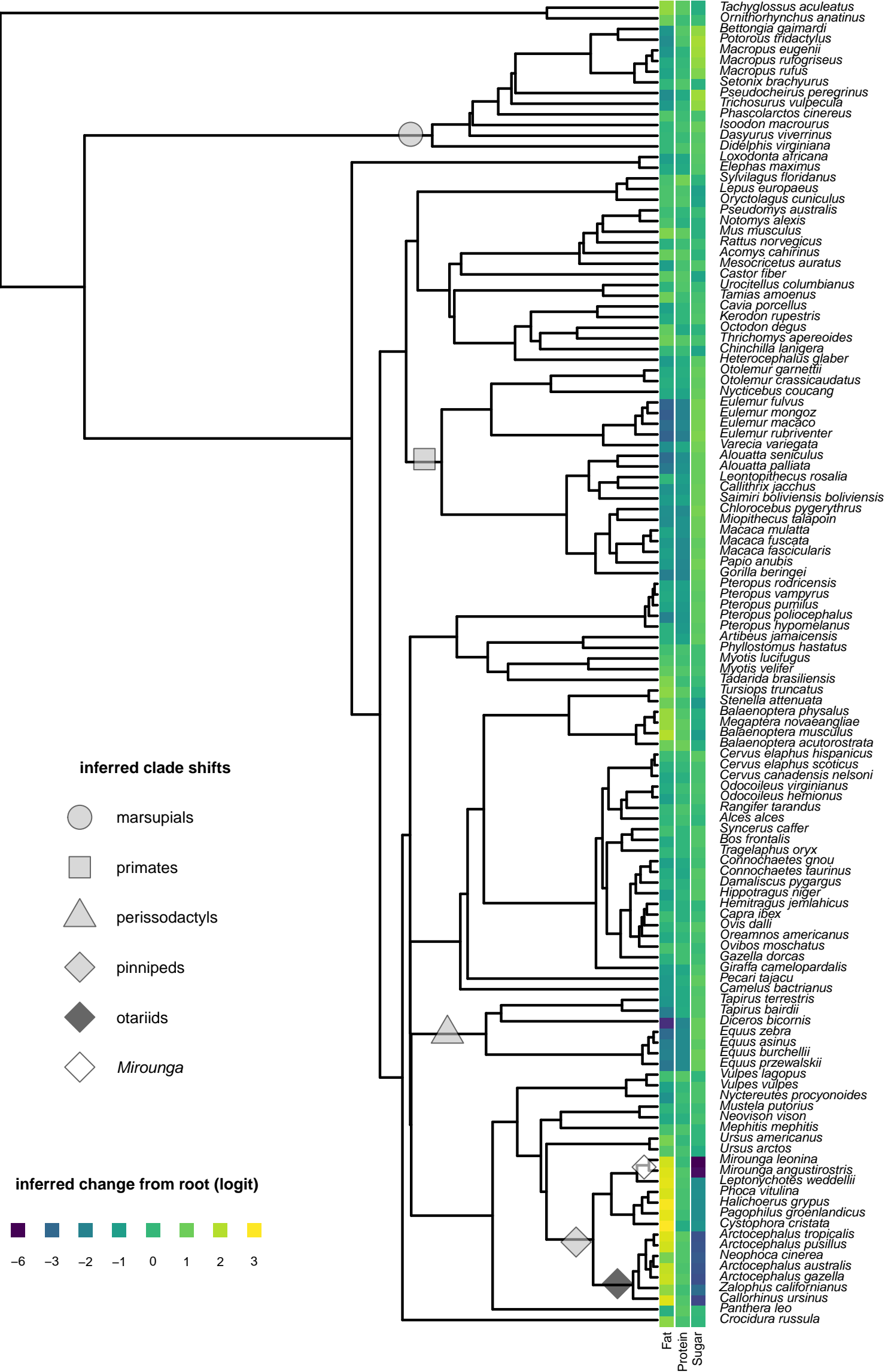

Supplement: Supplemental Information 1 [file peerj-07-8085-s001.zip › output/PhyloEMshifts.pdf]

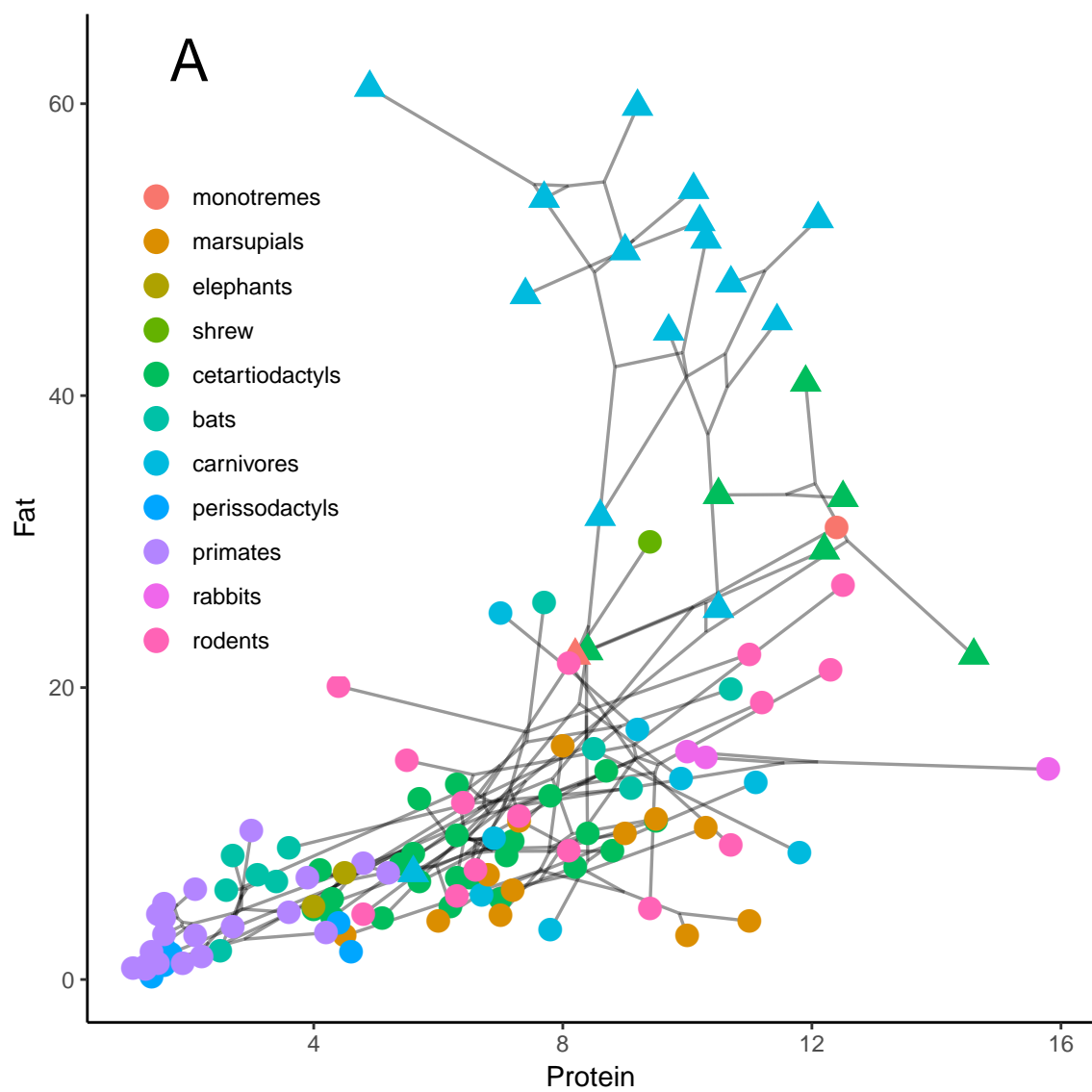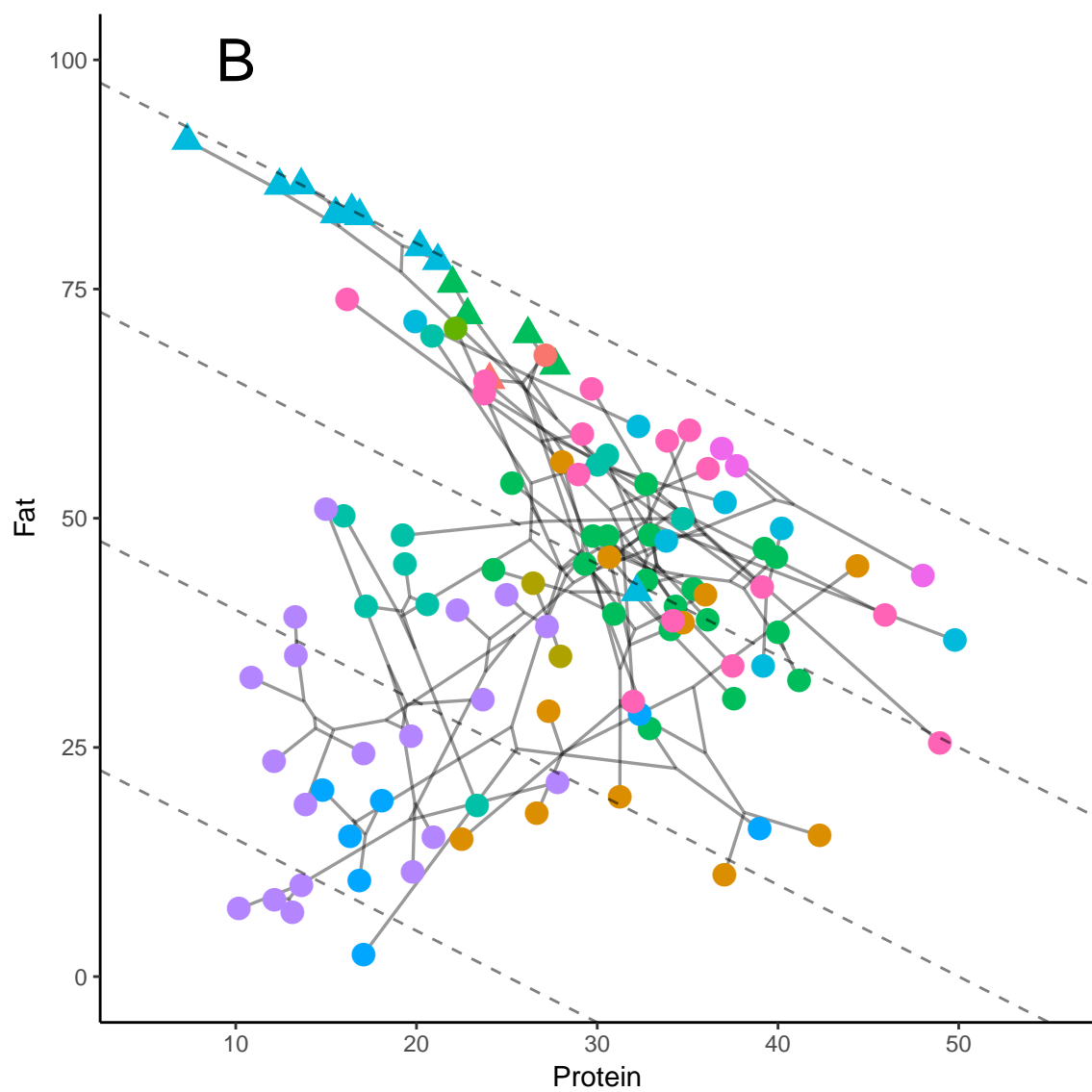

Supplement: Supplemental Information 1 [file peerj-07-8085-s001.zip › output/gPhylomorphoNutrGeom.pdf]

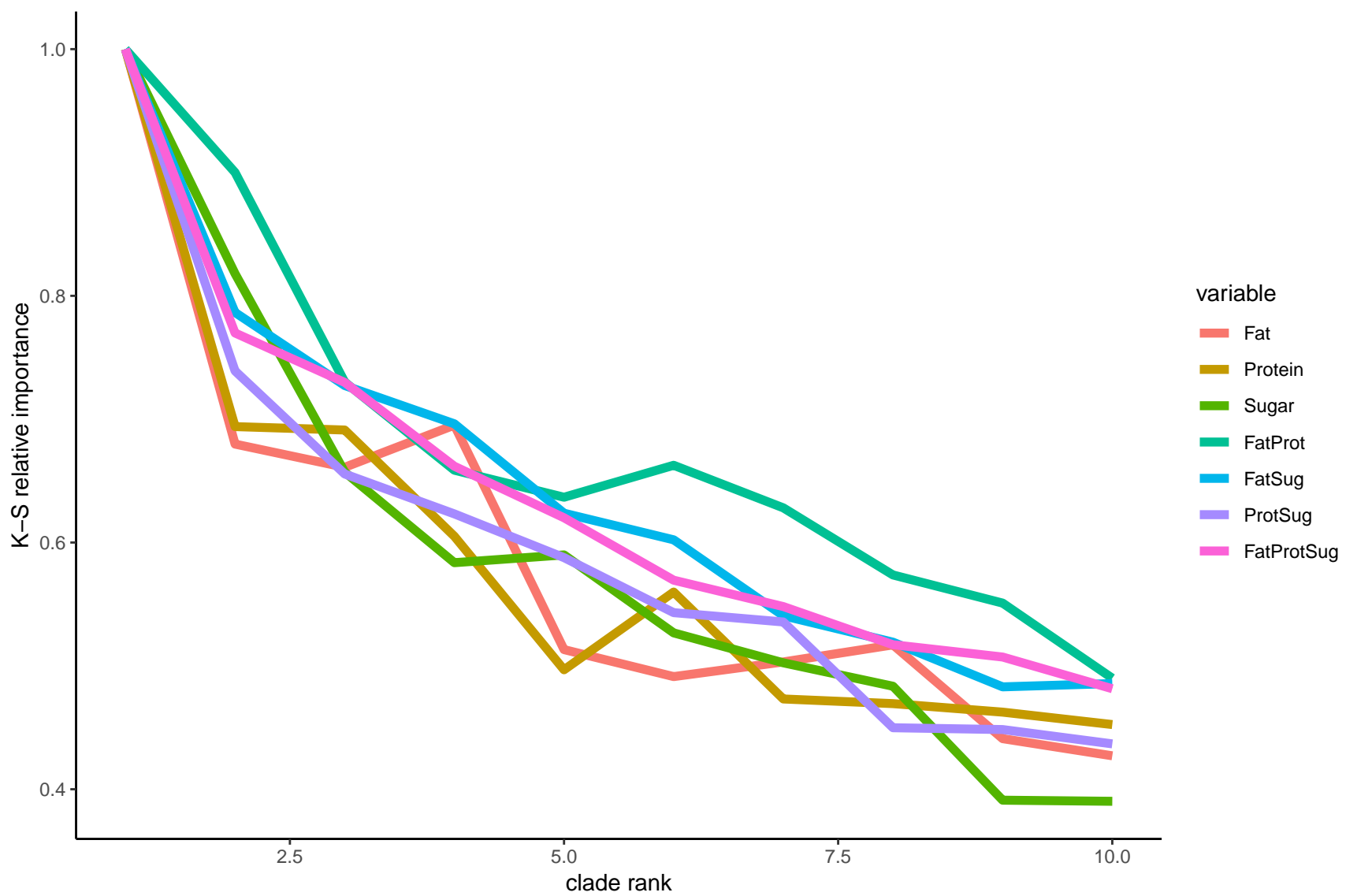

Supplement: Supplemental Information 1 [file peerj-07-8085-s001.zip › output/ksim.pdf]

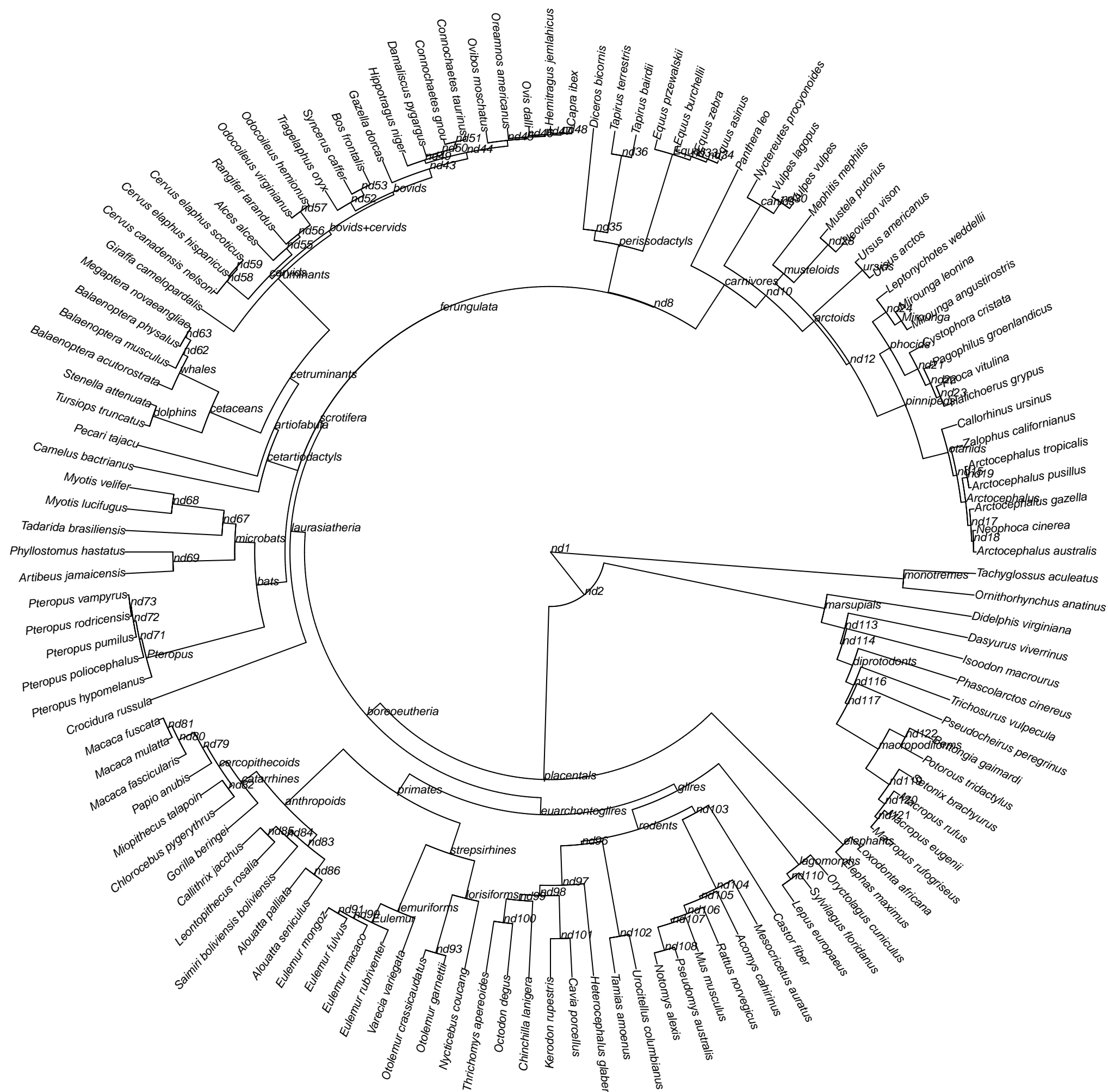

Supplement: Supplemental Information 1 [file peerj-07-8085-s001.zip › output/phylowithnodelabels-fan.pdf]
